# Phosphorylation of FOXK2 at Thr13 and Ser30 by PDK2 sustains glycolysis through a positive feedback manner in ovarian cancer

**DOI:** 10.1038/s41388-024-03052-x

**Published:** 2024-05-11

**Authors:** Cancan Zhang, Yinyin Xu, Xinyue Zhu, Xueli Zhang, Fengmian Wang, Lipeng Hu, Huan Lu, Chunlin Tao, Kai Xu, Zhigang Zhang, Dongxue Li, Tingyan Shi, Rong Zhang

**Affiliations:** 1 Fengxian Hospital, The Third School of Clinical Medicine, Southern Medical University, Shanghai, China; 2 Shanghai Geriatric Medical Center, Shanghai, 201104 China; 3 Department of Obstetrics and Gynecology, Ruijin Hospital, Shanghai Jiao Tong University School of Medicine, Shanghai, China; 4 State Key Laboratory of Oncogenes and Related Genes, Shanghai Cancer Institute, Renji Hospital, Shanghai Jiao Tong University School of Medicine, Shanghai, China; 5 Department of Gynecologic Oncology, Zhongshan Hospital, Fudan University, Shanghai, China

**Keywords:** Ovarian cancer, Cancer metabolism

## Abstract

Ovarian cancer is one of the most common gynecological malignant tumors with insidious onset, strong invasiveness, and poor prognosis. Metabolic alteration, particularly aerobic glycolysis, which is tightly regulated by transcription factors, is associated with the malignant behavior of OC. We screened FOXK2 in this study as a key transcription factor that regulates glycolysis in OC. FOXK2 is overly expressed in OC, and poor prognosis is predicted by overexpression. FOXK2 promotes OC cell proliferation both in vitro and in vivo and cell migration in vitro. Further studies showed that PDK2 directly binds to the forkhead-associated (FHA) domain of FOXK2 to phosphorylate FOXK2 at Thr13 and Ser30, thereby enhancing the transcriptional activity of FOXK2. FOXK2 transcriptionally regulates the expression of PDK2, thus forming positive feedback to sustain glycolysis in OC cells.

## Introduction

Ovarian cancer (OC) is the leading cause of death for gynecologic malignancies worldwide, mainly due to diagnosis at an advanced stage and chemotherapy resistance after high-rate recurrence [1]. Due to receiving nonstandard treatment or no treatment at all and chemotherapy resistance, some patients still fail to achieve desirable progression-free survival (PFS) [2]. Clarifying the mechanism of OC tumorigenesis and exploring potential therapeutic targets are essential to improve the prognosis of patients with OC.

The reprogramming of energy metabolism has been considered as a new hallmark of cancers while aerobic glycolysis has been regarded as the major metabolic phenotype [3]. Altered metabolic pathways in ovarian cancer facilitate cancer cell survival and proliferation while also influencing the ability to metastasize, acquiring resistance to chemotherapy, maintaining the cancer stem cell phenotype and escaping from the effects of antitumor immune defense [4]. Cancers and other proliferating cells exhibit increased expression of many glycolytic enzymes, which is consistent with their high utilization of glycolysis [5–7]. The glycolytic pathway contains 10 chemical reaction steps, each catalyzed by a specific enzyme. The overexpression of GLUT-1, which functions as a rate-limiting step in the flux of glucose for glycolysis, was associated with growth and poor prognosis in several human tumors [8, 9]. High ALDOA expression has been shown to boost glycolysis to promote the proliferation and invasion of cancer cells [10]. Cancer cells can significantly increase glucose uptake and utilization to enhance aerobic glycolysis, and thereby rapidly produce ATP and biosynthesis, ultimately promoting tumorigenesis and metastasis [11]. Simultaneously, a series of signaling pathways, including the PI3K/AKT, mTOR, MAPK, Wnt and AMPK signaling pathway, are involved in regulating aerobic glycolysis in cancer cells [12–15].

P53, c-myc, HIF-1 and SIX are transcription factors that play an important role in regulating the Warburg effect [16, 17]. C-myc enhances aerobic glycolysis by directly regulating glycolytic genes, including GLUTs, HK, PFK, PGK, enolase, and LDHA [18]. HIF-1α induces the glycolytic enzymes, including HKII, PFK1, LDHA, aldolase, and GLUT-1/3, to switch the glucose metabolism to the glycolytic pathway in hypoxic tumor cells [19].

The transcription factor-forkhead box K2 (FOXK2) belongs to the forkhead box (FOX) family, shares a conserved DNA binding domain and regulates a wide spectrum of biological processes within the cell. Regarded as an interleukin enhancer binding factor, FOXK2 was initially confirmed as a nuclear factor of an activated T-cell-like interleukin-binding factor. FOXK2 acts as a suppressor in breast cancer, renal clear cell carcinoma, non-small cell lung cancer, and glioma [20–23] but has oncogenic roles in colon cancer, colorectal cancer, and hepatocellular carcinoma [24–26]. A recent study has found that FOXK2 promotes ovarian cancer stemness by regulating the unfolded protein response pathway [27], and it regulate glycolysis in adipocyte [28], but the role of FOXK2 in glycolysis in OC is still unclear.

In this study, we identified FOXK2 as a key transcription factor regulating glycolysis in OC. FOXK2 was highly expressed in OC and played a tumor-promoting role. Pyruvate dehydrogenase Kinase2(PDK2) was shown in further studies to promote the phosphorylation of FOXK2 at serine and threonine, and enhance the transcriptional activity of FOXK2. Additionally, FOXK2 directly transcriptionally regulates the expression of PDK2. Therefore, PDK2-FOXK2 formed positive feedback to further increase the expression level of glycolytic enzymes and ultimately sustained glycolysis levels in OC cells.

## Results

### FOXK2 is screened as a key transcription factor that regulates glycolysis in OC

**Fig. 1:**
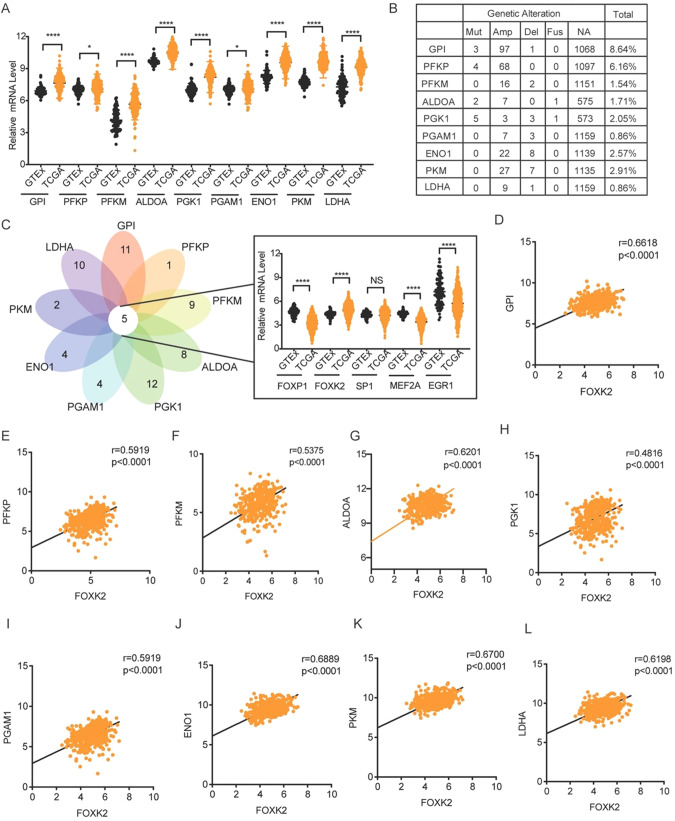
FOXK2 was screened as a key transcription factor that regulates glycolysis in OC. **A** The mRNA expression of nine key glycolytic enzymes in normal ovary from GTEx (*n* = 88) and ovarian cancer from TCGA (*n* = 428). **B** Genetic alteration of nine key glycolytic enzymes from public databases in the cBioPortal for Cancer Genomics. **C** Prediction of the transcription factors of nine key glycolysis enzymes through the JASPAR website. The shared five transcription factors of the top 50% high score transcription factors of nine key glycolytic enzymes and their mRNA expression in normal ovary from GTEx (*n* = 88) and ovarian cancer from TCGA (*n* = 428). **D**–**L** The correlation analysis of FOXK2 and 9 key glycolytic enzymes in OC samples (*n* = 428) from TCGA. Data are presented as the means ± SEM. *P < 0.05; ***P < 0.001, ****P < 0.0001.

We analyzed normal ovarian data from The Genotype-Tissue Expression (*GTEx*) and OC data from The Cancer Genome Atlas (TCGA) to investigate the expression of key glycolytic enzymes in OC. The nine key glycolytic enzymes exhibited high expression in OC compared with normal samples (Fig. 1A). We analyzed their genetic alterations in OC samples using the cBio Cancer Genomics Portal (cBioPortal) to further explore the causes of the increased expression of key glycolytic enzymes. Except for genomic alterations rates of GPI and PFKP exceeding 5%, the remaining 7 genes are all less than 3%. So, we speculated that their increased expression might be caused by transcription factors (Fig. 1B).

We used the JASPAR database to analyze all the respective transcription factors and took the top 50% of transcription factors for the intersection to determine which transcription factors could regulate all nine key glycolytic enzymes. Finally, five transcription factors were screened to regulate nine key enzymes simultaneously, of which only FOXK2 was highly expressed in OC (Fig. 1C, D). The correlation between FOXK2 and nine key glycolytic enzymes was further analyzed. As expected, FOXK2 was positively correlated with nine key glycolytic enzymes (Fig. 1E). We believe that FOXK2 is a key transcription factor regulating ovarian cancer glycolysis based on the above results.

### FOXK2 is upregulated in OC and is closely related to poor prognosis in OC patients

**Fig. 2:**
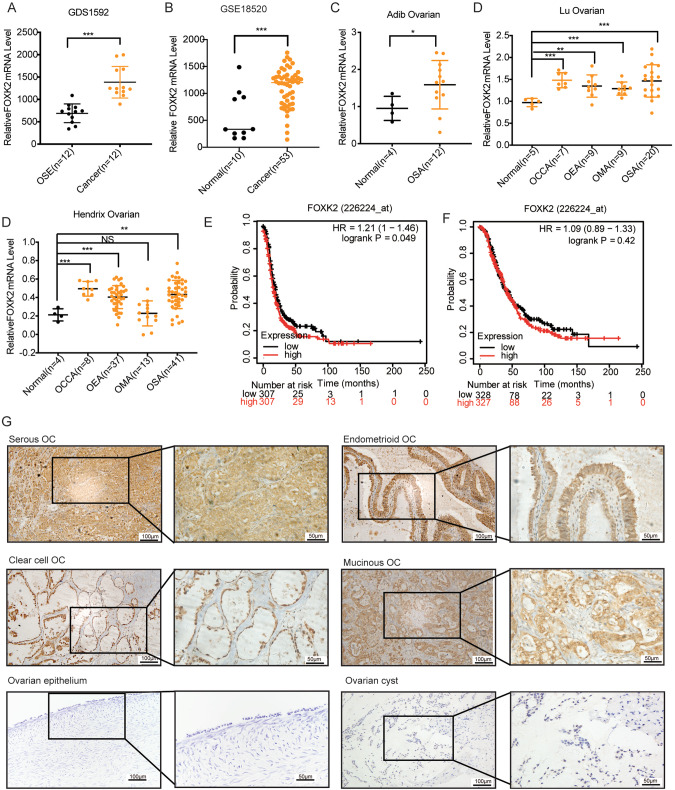
FOXK2 is upregulated in OC and is closely related to poor prognosis in OC patients. **A**–**D** FOXK2 expression analysis in tumors and normal tissues using Oncoming. OSE Ovarian surface epithelium, OCC Ovarian clear cell adenocarcinoma, OEA Ovarian endometrioid adenocarcinoma, OMA Ovarian mucinous adenocarcinoma, OSA Ovarian serous adenocarcinoma. Kaplan–Meier analysis of the progression-free survival (PFS, **E**) and overall survival (OS, **F**) of OC patients according to FOXK2 mRNA expression, generated based on GEO cohorts in Kaplan-Meier Plotter. The median PFS survival of low expression cohort is 19 months and the high expression cohort is 16 months and the median OS of low expression cohort is 45 months and the high expression cohort is 42 months. **G** Representative IHC images of FOXK2 in normal ovaries or ovarian cancer. Scale bar is 50 μm. Data are presented as the means ± SEM. *P < 0.05; **P < 0.01, ***P < 0.001, ****P < 0.0001.

We first analyzed FOXK2 expression using data from multiple databases to determine FOXK2 expression in OC and found that FOXK2 expression was significantly higher than that of the control individuals (Fig. 2A–E). We next performed Kaplan–Meier analysis to explore the relationship between FOXK2 expression and the prognosis of patients. The results suggest that high FOXK2 expression is closely related to poor prognosis in OC patients, as the average progression-free survival (PFS) in patients with high FOXK2 expression patients was shorter than that in patients with low FOXK2 expression (Fig. 2F).

We analyzed the FOXK2 expression status with respect to various pathological parameters in 191 OC patients to further investigate the clinical significance of FOXK2 in OC. The results indicated that FOXK2 expression was closely correlated with TNM stage and lymph node metastasis in OC tissues (Fig. 2G, Table 1). Overall, these results demonstrated that OC-upregulated FOXK2 acted as an indicator of OC progression and predicted poor prognosis.

**Table 1:** Correlation of the clinicopathological parameters with FOXK2 expression.

Catalog	Level	FOXK2 low	FOXK2 high	Total	*Χ*^2^	*P*
Classification						
	Ovarian cancer	86	105	191	37.702	0.000
	Benign cyst	55	10	65		
Age	≤50	33	42	75	0.053	0.819
	å 50	53	63	116		
Type	Serous OC	58	71	129	0.841	0.840
	Mucinous OC	9	14	23		
	Clear cell OC	13	12	25		
	Endometrial OC	6	8	14		
Stage	I	42	33	75	6.771	0.034
	II	11	13	24		
	III + IV	33	59	92		
Lymph node metastasis	Positive	14	33	47	5.849	0.016
	Negative	72	72	144		

### Inhibiting FOXK2 expression suppresses cell proliferation and migration in OC

**Fig. 3:**
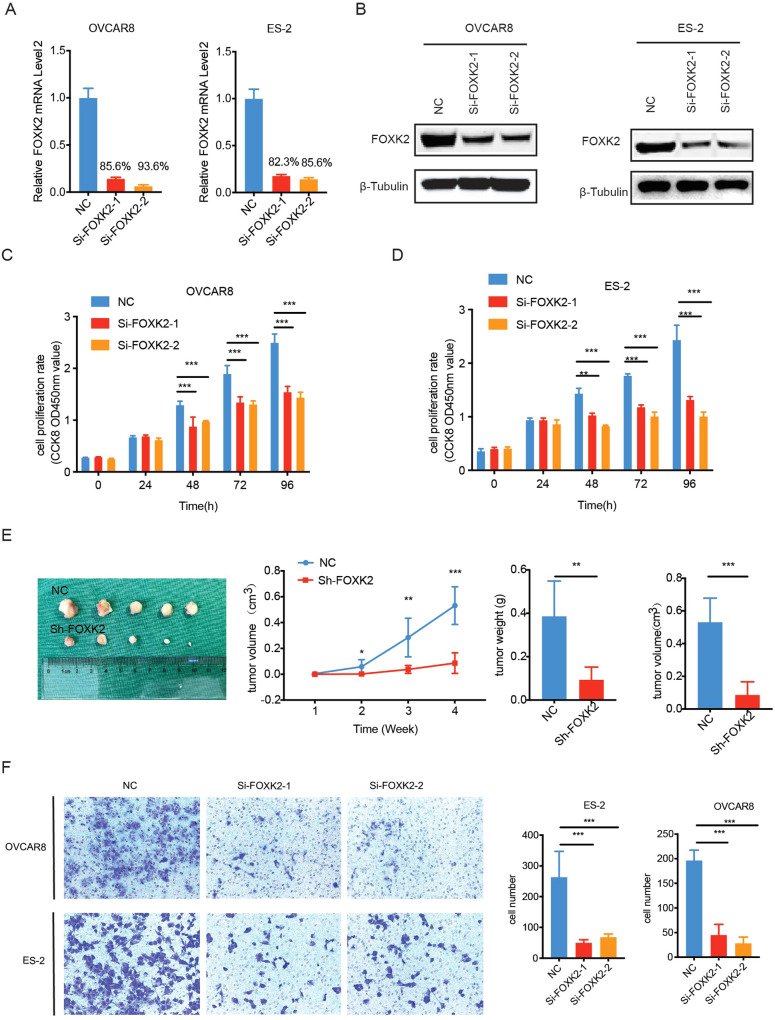
Inhibiting FOXK2 expression suppresses the cell proliferation and migration in OC. **A**, **B** Validation of FOXK2 interference efficiency at protein and mRNA levels in OVCAR8 and ES-2 cells. **C**, **D** A CCK-8 assay was conducted using NC or si-FOXK2 in OVCAR8 and ES-2 cells. **E** Subcutaneous xenografts transplanted with OVCAR8 cells expressing shNC and shFOXK2 (*n* = 5). Left: tumor growth curve in vivo. Middle: tumor growth curve in vivo. Right: Tumor weight and volume. **F** Cellular migration ability was detected by transwell migration in OVCAR8 and ES- 2 cells expressing shNC or siFOXK2. Left: representative pictures; Right: the number of migrated cells. Data are presented as the means ± SEM. *P < 0.05; **P < 0.01; ***P < 0.001, ****P < 0.0001.

We first knocked down FOXK2 in OC cells and confirmed the efficiency (Fig. 3A, B) to determine the biological functions of FOXK2 in OC cells. Then we investigated the effects of FOXK2 on OC cell proliferation. FOXK2 knockdown obviously inhibited cell growth in vitro (Fig. 3C, D). A subcutaneous xenograft model was established to explore the impacts of FOXK2 expression on tumors in vivo. The results showed that knockdown of FOXK2 significantly reduced tumor growth (Fig. 3E).

We additionally investigated the effects of FOXK2 on OC cell migration. FOXK2 knockdown apparently inhibited cell migration (Fig. 3F). These results indicate that FOXK2 promotes the malignant phenotypic transformation of OC, suggesting that FOXK2 functions as an oncogene in OC.

### Inhibiting FOXK2 expression reduces the glycolysis levels in OC

**Fig. 4:**
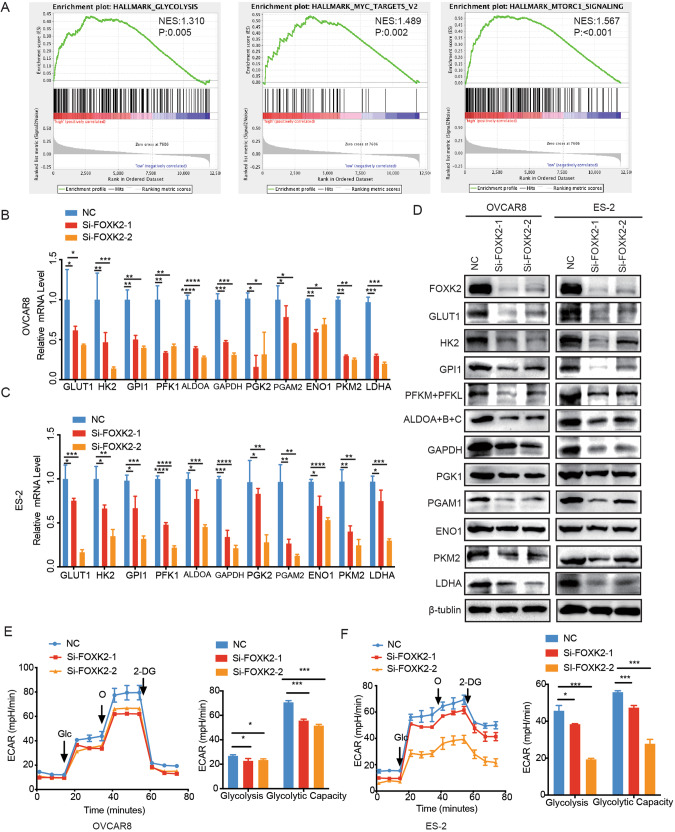
Inhibiting FOXK2 expression reduces the glycolysis levels in OC. **A** GSEA of specimens with high and low expression of FOXK2 based on the data from TCGA. Representative gene sets are upregulated in the FOXK2 high-phenotype. (NES, normalized enrichment score.). **B**, **C** Relative mRNA expression of key enzymes in glycolysis in control and FOXK2 knockdown OVCAR8 and ES-2 cells. **D** Relative protein expression of key enzymes in glycolysis in control and FOXK2 knockdown OVCAR8 and ES-2 cells. **E**, **F** Extracellular acid ratio (ECAR) upon knockdown by Si-FOXK2 or not in OVCAR8 and ES-2 cells (ECAR, *n* = 3). Glc glucose, O oligomycin, 2-DG 2-deoxy-glucose. Data are presented as the means ± SEMs. *P < 0.05; **P < 0.01; ***P < 0.001, ****P < 0.0001.

We first investigated whether FOXK2 affects glycolytic capacity in OC. FOXK2 was screened as a transcription factor that regulates several key enzymes of glycolysis. The OC samples in the TCGA database were divided into two groups by median FOXK2 expression. Striking alterations in metabolic processes, including glycolysis, Myc targets and mTORC1 signaling (Fig. 4A), were shown using the hallmark gene sets of two group transcriptome data using Gene Set Enrichment Analysis (GSEA).

We knocked down FOXK2 to further verify the effect of FOXK2 on the expression levels of key enzymes in glycolysis and then detected the RNA and protein levels of key enzymes. The results showed that the RNA and protein levels of 11 key enzymes were significantly reduced after FOXK2 knockdown (Fig. 4B–D).

We examined the extracellular acidification rate (ECAR; an indicator of glycolysis) with the Seahorse XF96 analyzer to verify the effect of FOXK2 on glycolysis levels in OC. As shown in Fig. 4E, F, we observed a reduction in the glycolytic rate (ECAR) following the FOXK2-siRNA treatment of OVCAR8 and ES-2 cells. Taken together, these data suggest that the tumorigenic effect by activation of the FOXK2 may be largely resulted from its enhancement of glycolysis.

### Transcription factor FOXK2 directly regulates 7 key enzymes of glycolysis in OC

To demonstrate whether FOXK2 could directly regulate the expression of key enzymes in glycolysis, we used MEME software to predict the binding site of FOXK2 to key enzymes and found that FOXK2 had binding sites with the promoter regions of 7 key enzymes, GPI, PKM2, PGK1, GAPDH, LDH, PFK and HK2. The databases of ChIP Seq also showed that these key enzymes were targets of FOXK2 (Supplementary Table S4).

**Fig. 5:**
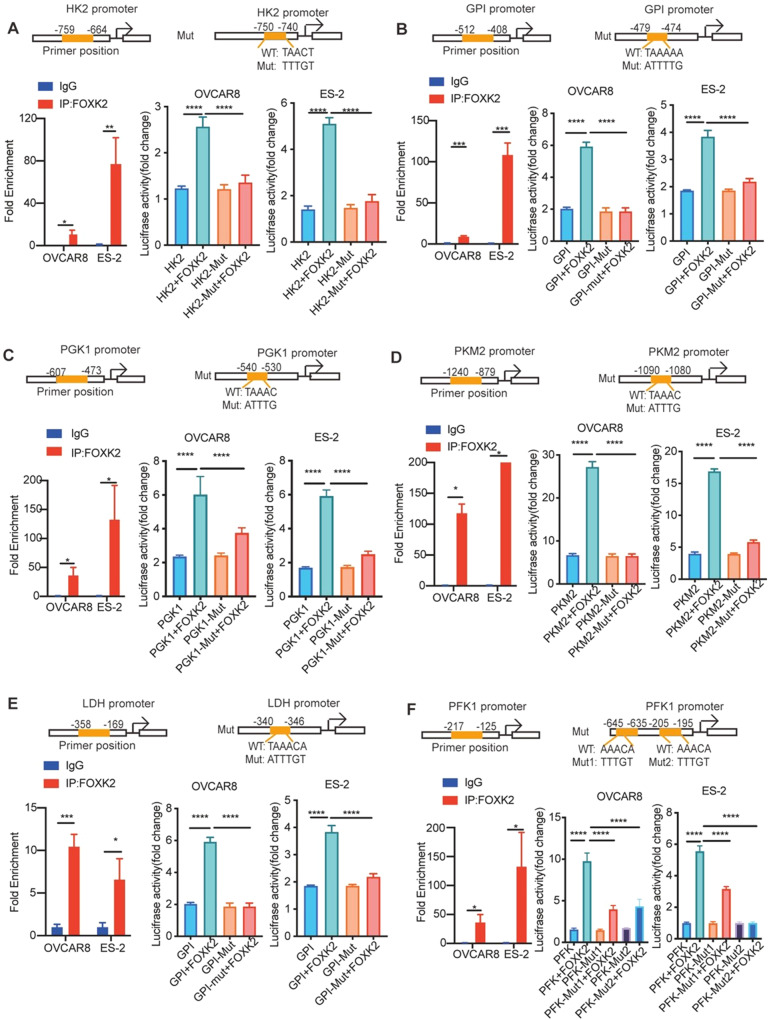
The transcription FOXK2 directly regulates six key enzymes of glycolysis in OC. **A**–**F** ChIP assays using the FOXK2 antibody followed by qPCR of the promoter region of 6 key enzymes in OVCAR8 and ES-2 cells. The binding sites of FOXK2 to the promoter region of 6 key enzymes predicted by MEME and luciferase reporter assay was performed using promoter in OVCAR8 and ES-2 cells after transfecting the wild type plasmids and mutated plasmids (mutation site: orange). Data are presented as the means ± SEMs. *P < 0.05; ***P < 0.001, ****P < 0.0001.

We constructed the primers according to the promoters of the six key enzymes, and we performed ChIP PCR in OVCAR8 and ES-2 cells to evaluate whether FOXK2 could directly bind to the promoters. As expected, ChIP analysis indicated that FOXK2 directly bound to specific regions in the 7 key enzyme promoters. (Fig. 5A–F and Supplementary Fig. S1). Luciferase reporter assays further confirmed that the transcriptional activity of the GPI, PKM2, PGK1, GAPDH, LDH, PFK and HK2 promoters was significantly induced by FOXK2 (*P* < 0.001) and was significantly decreased by the seven above promoter mutations (Fig. 5A–F and Supplementary Fig. S1). These data suggest that FOXK2 directly regulates the expression of HK2, GPI, PKM2, PGK1, GAPDH, LDH, and PFK at the transcriptional level in OC cells.

### PDK2 phosphorylates FOXK2 at serine and threonine and promotes FOXK2 transcriptional activity

**Fig. 6:**
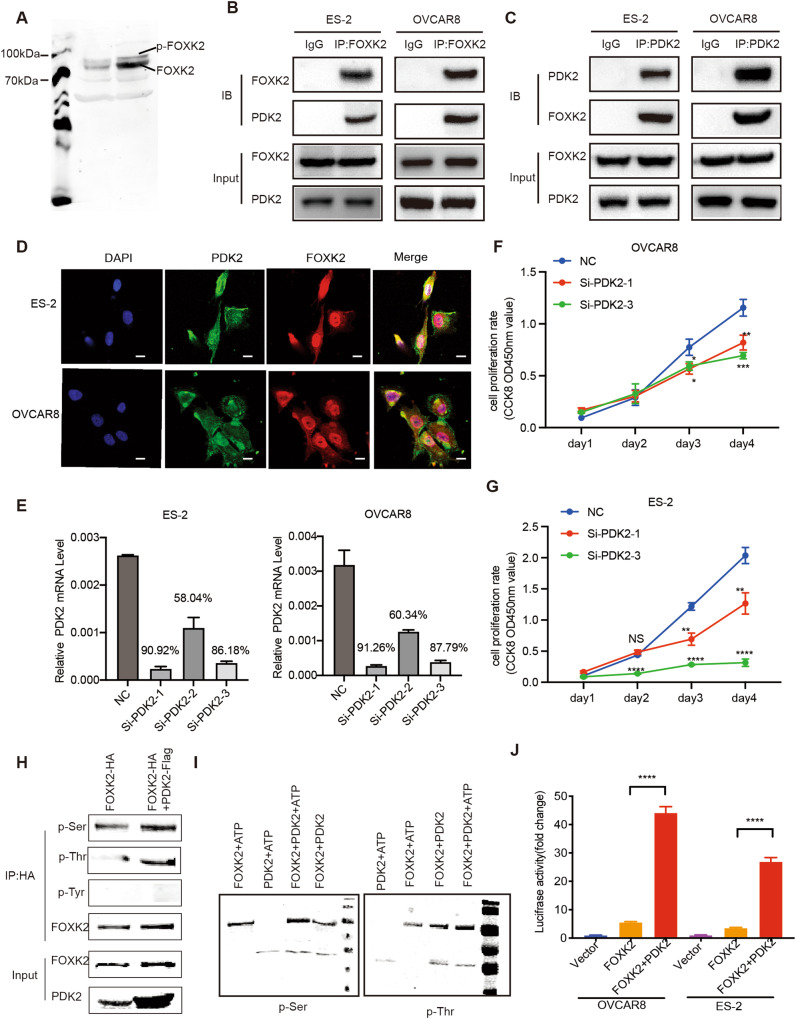
PDK2 phosphorylates FOXK2 at serine and threonine and promotes FOXK2 transcriptional activity. **A** Western blotting was used to detect the phosphorylation of FOXK2 using the phos-tag gels. **B**, **C** Co-immunoprecipitation of PDK2 and FOXK2 in OVCAR8 and ES-2 cells. **D** Immunofluorescence of PDK2 and FOXK2 in OVCAR8 and ES-2 cells. Scale bar is 10 μm. **E** Interference efficiency verification of PDK2 in OVCAR8 and ES-2 cells. **F**, **G** Relative cell viability of OVCAR8 and ES-2 cells expressing NC or si-PDK2. **H** Detection of serine, threonine, and tyrosine phosphorylation in purified FOXK2 protein by immunoprecipitation using western blotting. **I** Detection of serine and threonine phosphorylation in in vitro kinase reaction products by western blotting. **J** Luciferase reporter assay was performed using the HK2 promoter in OVCAR8 and ES-2 cells after transfecting the wild type plasmids (Vector), FOXK2 and PDK2 plasmids. Data are presented as the means ± SEMs. *P < 0.05; **P < 0.01, ***P < 0.001, ****P < 0.0001.

Protein phosphorylation is one of the most common, yet key posttranslational modifications that induce conformational changes in proteins. By analyzing the data in PhosphoSitePlus, a comprehensive resource for investigating the structure and function of experimentally determined post translational modifications in human and mice, we discovered many phosphorylation sites in the FOXK2 protein (Supplementary Fig. S2). We used Phosbind acrylamide to separate phosphorylated and nonphosphorylated proteins and demonstrated the presence of phosphorylated of FOXK2 in OC cell proteins by western blotting (Fig. 6A) due to the lack of FOXK2 phosphorylated antibodies.

We queried the BioGRID interaction database to identify kinases that phosphorylate FOXK2 and found that the kinase PDK2 may interact with FOXK2. To verify the interaction between FOXK2 and PDK2, we conducted CO-IP experiments in OVCAR8 and ES-2 cells. The results showed that FOXK2 could interact with PDK2 (Fig. 6B, C). Immunofluorescence of FOXK2 and PDK2 additionally displayed obvious colocalization in the nucleus and a small amount in the cytoplasm (Fig. 6D). To characterize the effect of PDK2 on the tumorigenicity of OC cells, we knocked down the expression of PDK2 in OVCAR8 and ES-2 cells (Fig. 6E). In contrast to control cells, the cell proliferation ability of PDK2-silenced cells was significantly reduced (Fig. 6F, G).

To determine whether PDK2 phosphorylates FOXK2, we first performed FOXK2 single transfection or FOXK2/PDK2 double transfection in OC cells to detect the phosphorylation type of FOXK2 protein by PDK2. We performed immunoprecipitation on the protein extracts and found that the FOXK2 protein was phosphorylated by serine and threonine, but no by tyrosine (Fig. 6H). Then, we performed in vitro kinase assay to determine whether PDK2 phosphorylates FOXK2 using their purified protein. The results showed that the amount of ATP remaining in solution following a kinase reaction was only approximately 8% left (Supplementary Fig. S3). The serine/threonine band of FOXK2 was significantly increased in the presence of ATP (Fig. 6I), demonstrating that this reaction consumed a large amount of ATP and that PDK2 facilitated serine and threonine phosphorylation of FOXK2. These data suggest that FOXK2 is a novel protein substrate of PDK2 and can be phosphorylated at serine and threonine residues.

We previously reported that FOXK2 binds to the promoters of glycolysis-related genes. To further explore the effect of PDK2 on the transcriptional activity of FOXK2, we performed luciferase reporter assays and found that overexpression of PDK2 enhanced GPI promoter activity (Fig. 6J). These data suggest that PDK2 promotes FOXK2 transcriptional activity.

### FOXK2 Thr13 and Ser30 phosphorylation by PDK2 increases FOXK2 transcriptional activity

**Fig. 7:**
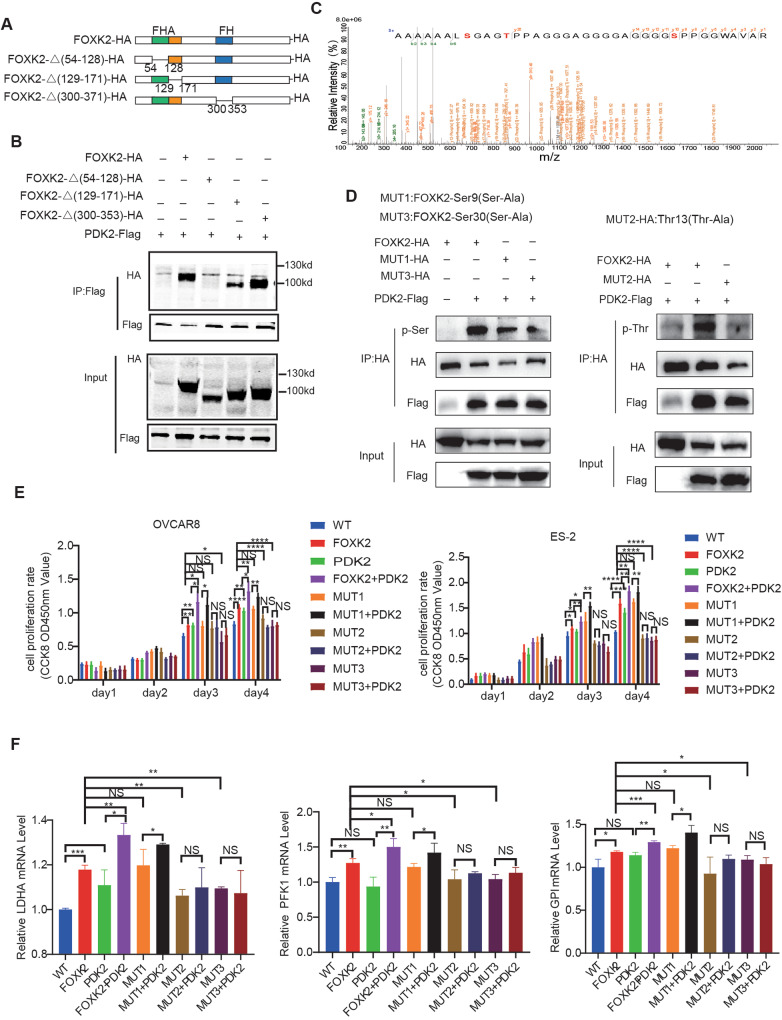
FOXK2 Thr13 and Ser30 phosphorylation by PDK2 increases FOXK2 transcriptional activity. **A**, **B** Schematic diagram of truncation of FOXK2 according to the FOXK2 domain and Co-immunoprecipitation of PDK2 with FOXK2 truncations. **C** The spectrum of the 38-residue-long peptide (Start position is 2 and end position is 39 in FOXK2 protein). **D** Detection of serine and threonine phosphorylation in purified FOXK2 protein by immunoprecipitation using western blotting. **E** Relative cell viability OVCAR8 and ES-2 expressing single or both FOXK2, PDK2 and mutant plasmids in OVCAR8 and ES-2 cells. **F** Relative mRNA expression of FOXK2 targets in single or both FOXK2, PDK2 and mutant plasmids-transfected OVCAR8 and ES-2 cells. Data are presented as the means ± SEMs. *P < 0.05; **P < 0.01, ***P < 0.001, ****P < 0.0001.

We truncated the protein domain of FOXK2 to determine the specific binding site of PDK2 to FOXK2, and then co-immunoprecipitated PDK2 with the protein truncation of FOXK2. The results are shown in the Fig. 7A, B. PDK2 could interact with the truncated proteins without the second or third domains but not with the truncated proteins without the first domain-FHA, indicating that PDK2 specifically bound the FHA domain of FOXK2.

Then we performed mass spectrometry (MS) analysis of the protein products after the kinase reaction to confirm the specific phosphorylation sites, and found three sites, Ser9, Thr13, and Ser30, were significantly phosphorylated (Fig. 7C, Supplementary Table S5), and these three sites have been predicted on the website PhosphoSitePlus (Supplementary Fig. S2). Next, we generated the original plasmid and phosphorylation site mutant plasmids and performed FOXK2 single transfection, FOXK2/PDK2 or FOXK2 mutant /PDK2 double transfection in OC. We performed immunoprecipitation on the protein extracts and found that serine and threonine phosphorylation were significantly enhanced in FOXK2-PDK2 double-transfected cells but reduced in FOXK2 mutant-PDK2 double-transfected cells. These results indicate that PDK2 phosphorylates FOXK2 at Ser9, Thr13, and Ser30.

We further examined whether phosphorylation of FOXK2 at Ser9, Thr13, and Ser30 enhances ovarian cancer cell proliferation. The proliferation assay results showed that FOXK2 and PDK2 alone enhanced cell proliferation compared with the vector group; of note, cell proliferation was inhibited by the Thr13 and Ser30 mutants compared with FOXK2, while the Ser9 mutant promoted cell proliferation at a similar level to FOXK2 (Fig. 7E). Next, we investigated the effect of PDK2 on cancer cell growth through phosphorylation of FOXK2 at Ser9, Thr13 or Ser30 and found that FOXK2 or Ser9-mediated induction of cancer cell proliferation could be induced by PDK2 overexpression in cells. However, such proliferation enhancement was attenuated in cells expressing Thr13 and Ser30 even in the presence of PDK2 overexpression (Fig. 7E). In addition, we overexpressed FOXK2 or mutants in cells that interfered with FOXK2, and the results showed that FOXK2 or Ser9A rescued the knockdown-induced suppression of proliferation, but not in Ser30A or Thr13A (Supplementary Fig. S4A, B).

At the same time, we examined the mRNA expression of FOXK2 targets in OC cells expressing FOXK2 or serine and threonine mutants. As expected, the mRNA expression level of FOXK2 targets was increased upon FOXK2 or Ser9 mutant overexpression, whereas cells expressing phosphorylation Thr13 and Ser30 mutants could not upregulate FOXK2 targets (Fig. 7F). Next, we investigated the effect of PDK2 on the mRNA expression of FOXK2 targets and found that the mRNA expression level of FOXK2 targets could be induced by PDK2 overexpression in cells. However, this enhancement was attenuated in cells expressing Thr13 and Ser30 even in the presence of PDK2 overexpression (Fig. 7F). These data indicate that PDK2 phosphorylates FOXK2 at Thr13 and Ser30 lead to an increase in the mRNA level of the target genes, which may be due to PDK2 promoting transcriptional activation of FOXK2.

### FOXK2 Thr-13 and Ser-30 phosphorylation by PDK2 promotes the glycolytic ability of OC cells

**Fig. 8:**
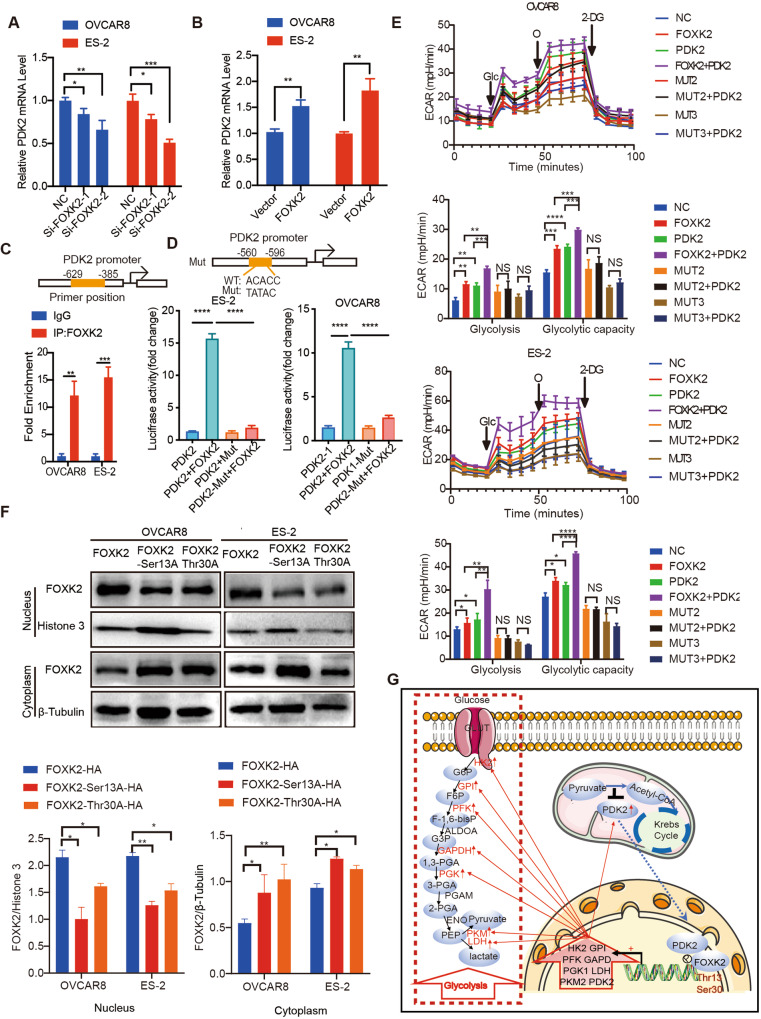
FOXK2 directly transcriptional regulates PDK2 in OC. **A** Relative mRNA expression of *PDK2* in control and FOXK2 knockdown OVCAR8 and ES-2 cells. **B** Relative mRNA expression of *PDK2* in empty vector and FOXK2-overexpressing OVCAR8 and ES-2 cells. **C**, **D** ChIP assays using the FOXK2 antibody followed by qPCR of the promoter region of PDK2 in OVCAR8 and ES-2 cells. The binding sites of FOXK2 to the promoter region of PDK2 predicted by MEME and luciferase reporter assays were performed using PDK2 the promoter in OVCAR8 and ES-2 cells after transfecting the wild type plasmids and mutated plasmids (mutation site: orange). **E** Extracellular acid ratio (ECAR) upon overexpression of single or both FOXK2, PDK2 and mutant plasmids in OVCAR8 and ES-2 cells (ECAR, *n* = 3). Glc glucose, O oligomycin, 2-DG 2-deoxy-glucose. **F** The expression of FOXK2 in cytoplasm and nucleus of FOXK2 and mutant in OVCAR8 and ES-2 cells. FOXK2-T13A: Thr13 (Thr-Ala) ; FOXK2-S30A: Ser30 (Ser-Ala). **G** Proposed model for PDK2-FOXK2 sustaining glycolysis through a positive feedback manner in ovarian cancer. Data are presented as the means ± SEMs. *P < 0.05; ***P < 0.001, ****P < 0.0001.

Through analysis of our RNA-seq data, we found that PDK2 may be the target of FOXK2. Therefore, we knocked down or overexpressed FOXK2 to verify the effect of FOXK2 on the expression levels of PDK2 and found that the mRNA of PDK2 was significantly reduced after FOXK2 knockdown and was significantly increased after FOXK2 overexpression (Fig. 8A, B).

To demonstrate whether FOXK2 directly regulate the PDK2 expression, we performed ChIP PCR and luciferase reporter assays. ChIP analysis indicated that FOXK2 directly bound to the promoter region of PDK2 (Fig. 8C). Luciferase reporter assays further confirmed that the transcriptional activity of the PDK2 promoter was significantly induced by FOXK2 and was significantly decreased by promoter mutation (Fig. 8D).

To explore the effect of FOXK2 Thr-13 and Ser-30 phosphorylation on glycolysis, we examined the ECAR with the Seahorse XF96 analyzer and found that the glycolysis level was increased upon FOXK2 or PDK2 overexpression, whereas cells expressing phosphorylated mutants (T13A and S30A) could not upregulate the glycolysis level. Additional increase in PDK2 expression resulted in a further increase in glycolytic capacity in the FOXK2 group, while the mutant group did not show a significant increase (Fig. 8E). In addition, we overexpressed FOXK2 or mutants in cells that interfered with FOXK2, and the results showed that FOXK2 or Ser9A rescued the knockdown-induced suppression of glycolysis, but not in Ser30A or Thr13A (Supplementary Fig. S4C, D). These data indicate that PDK2 phosphorylates FOXK2 at Thr13 and Ser30 to promote the glycolytic ability of OC cells.

To explore whether phosphorylation sites at the Thr13 and Ser30 affect the translocation of FOXK2, we transfected FOXK2 and phosphorylated mutants (T13A and S30A) into OC cells, and then performed nuclear and cytoplasmic protein extraction to detect subcellular localization changes of FOXK2 protein. The results showed that after Thr13 and Ser30 mutation, the localization of FOXK2 in the nucleus significantly decreased compared to that in the nonmutated FOXK2 expression group (Fig. 8F), indicating that the phosphorylation of Thr13 and Ser30 was responsible for the translocation of FOXK2 into the nucleus for its effect on the transcription of glycolytic enzymes. In conclusion, our research found that FOXK2 directly regulates multiple genes of the glycolysis pathway through transcriptional regulation, thereby promoting the glycolysis in OC. Phosphorylation of FOXK2 at Thr13 and Ser30 by PDK2 promotes the nuclear translocation of FOXK2, thereby promoting its transcriptional regulation. In addition, high expression of FOXK2 also directly regulated the expression of PDK2 by transcription, and thus formed positive feedback to sustain in OC cell glycolysis, thereby promoting OC progression (Fig. 8G).

## Discussion

The reprogramming of energy metabolism has been considered as a new hallmark of cancers while aerobic glycolysis has been regarded as the major metabolic phenotype. Several enzymes in the glycolytic pathway, such as GLUT1, HK2, PKM2 and LDHA, have been identified as promising therapeutic targets for anticancer intervention and numerous studies have shown that the proliferation and progression of OC cells are dependent on glycolysis [29, 30]. In our study, we found that the expression of all enzymes was significantly increased in OC without apparent copy number alterations. We therefore considered that the high expression of glycolytic genes may be caused by upstream transcription factors. Further study revealed that these glycolytic enzymes were all transcriptionally regulated by FOXK2. FOXK2 was identified as a key factor regulating glycolysis in OC.

FOXK2 is a transcription factor belonging to the forkhead box (FOX) family. The function of FOXK2 in tumorigenesis has been characterized in several studies. FOXK2 was shown to promote tumor progression by activating Wnt signaling in colon cancer [25] but suppressed ER–positive breast cancer cell proliferation by interacting with transcriptional corepressor complexes [21]. Recently, it was reported that *FOXK2* was upregulated in ovarian CSCs and in human ovarian tumors and implicated in the regulation of the cellular stress response [27]. Our study suggests that FOXK2 is upregulated in the ovarian cancer, which was consistent with existing research results. Our further research showed that FOXK2 promoted cell proliferation and migration and that FOXK2 expression was related to tumor stage and lymph node metastasis in our clinical samples. FOXK2 may be a novel target in OC.

PDK is one of the key regulators of glycolysis and oxidative phosphorylation. PDK2 suppresses the conversion of pyruvic acid to acetyl coenzyme A, which enters the TCA cycle in mitochondria by inhibiting pyruvate dehydrogenase (PDH) through phosphorylation of Ser293 on the E1α subunit of PDH (PDHE1-alpha) [31]. PDK2 leads to cisplatin resistance in ovarian clear cell carcinoma through suppression of mitochondrial function [32]. FOXK2 is structurally characterized by a forkhead-associated (FHA) domain, a FOX domain and a nuclear localization signal. FHA domains are small phosphopeptide recognition modules found in eubacteria and eukaryotes that specifically recognize phosphorylated residues, particularly threonine and serine [33]. In our study, we were surprised to find that FOXK2 interacts with the kinase PDK2 and that PDK2 directly binds to the FHA domain of FOXK2 to phosphorylate FOXK2 at threonine and serine and PDK2 enhances the transcriptional activity of FOXK2. Therefore, we speculate that PDK2 regulates FOXK2 transcriptional activity by phosphorylating FOXK2 at serine and threonine.

The function of FOXK2 Is affected by epigenetic modifications. Posttranslational modifications (PTMs) of proteins link cellular signals to the functional properties. A ubiquitously utilized mechanism to transduce extracellular signals to the nucleus is rapidly reversible phosphorylation, which may affect transcription factor stability, location, structure, and/or the protein–interaction network. FOXK2 SUMOylation at the K527 and K633 sites modulates its transcriptional activity in response to paclitaxel. Paclitaxel resistance is associated with the inability of FOXK2 to bind target genes [34]. FOXK2 phosphorylation plays a role in defining FOXK2 function by regulating its stability and its activity as a transcriptional repressor protein at serine 368 and 423 sites by CDK [35]. Here, we reported PDK2 could phosphorylate FOXK2 at Thr13 and Ser30, and enhanced the transcriptional activity of FOXK2. In cells overexpressing FOXK2, additional expression of PDK2 significantly increased proliferation and glycolytic ability, but there was no similar effect in the FOXK2 Thr13 and Ser30 mutation groups. In addition, high expression of FOXK2 also directly regulates the expression of PDK2 by transcription. Moreover, after dephosphorylation mutation, the entry of FOXK2 into the nucleus is significantly reduced, affecting its transcriptional activity. Ultimately leading to a decrease in glycolytic capacity. Indicating that the phosphorylation site of FOXK2 regulated by PDK2 sustained glycolysis in ovarian cancer.

In adipocytes, overexpression of FOXK1 or FOXK2 upregulates PDK1 and PDK4, and Ser232 on the E1α subunit of PDKs was significantly positively regulated by overexpression or knockdown of FOXK2 [28]. In our study, we found the expression of PDK2 was significantly positively regulated by FOXK2, and FOXK2 transcriptionally regulates PDK2 directly. In ovarian cancer, the expression of PDK1 and PDK4 were relatively low compared to PDK2, so we haven’t further explored whether they can phosphorylate FOXK2 [28]. In our study, we found the expression of PDK2 was significantly positively regulated by FOXK2, and FOXK2 transcriptionally regulates PDK2 directly. In ovarian cancer, the expression of PDK1 and PDK4 were relatively low compared to PDK2, so we haven’t further explored whether they can phosphorylate FOXK2.

In conclusion, we identified FOXK2 as a key transcription factor that regulates OC glycolysis and provided a novel oncogenic regulatory mechanism of phosphorylation by PDK2 in regulating FOXK2 transcriptional activity. PDK2-mediated FOXK2 phosphorylation at Thr13 and Ser30 upregulated the expression of FOXK2 downstream glycolytic genes. FOXK2 transcriptionally regulates glycolytic genes directly, and FOXK2 phosphorylation by PDK2 enhances its transcriptional activity leading to upregulation of the expression of FOXK2 downstream genes, including glycolytic genes. In addition, high expression of FOXK2 also directly regulates the expression of PDK2 by transcription and thus orms positive feedback to sustain in OC cell glycolysis, thereby promoting the OC occurrence.

## Materials and methods

### Clinical samples and database

The human ovarian cancer and normal ovarian tissues used in this study were obtained from the Department of Obstetrics and Gynecology, Fengxian District Center Hospital and the Department of Obstetrics and Gynecology, The Affiliated Changzhou No. 2 People’s Hospital of Nanjing Medical University. None of the patients had received radiotherapy, chemotherapy, or other related antitumor therapies before surgery. All human tissues were obtained with informed consent, and the study was approved by the Research Ethics Committee of Fengxian District Center Hospital. We downloaded and analyzed the ovarian cancer cohorts in The Cancer Genome Atlas (TCGA, https://cancergenome.nih.gov/), GTEx (https://www.gtexportal.org/home/index.html) and the Gene Expression Omnibus (GEO, https://www.ncbi.nlm.nih.gov/geo/). The RNA-seq level 4 gene expression data contain log2-transformed RNA-seq by expectation maximization (RSEM) values summarized at the gene level. Genetic alteration data were downloaded from the cBio Cancer Genomics Portal (http://cbioportal.org/). Transcription factors of glycolytic genes were downloaded in JASPAR (https://jaspar.genereg.net).

### Cell culture and reagents

The human ovarian cancer cell lines OVCAR8 (RRID:CVCL_1629), ES-2 (RRID:CVCL_3509),human embryonic kidney 293T (RRID:CVCL_0063) cells were all preserved in Shanghai Cancer Institute, Ren Ji Hospital, School of Medicine, Shanghai Jiao Tong University. OVCAR8 cells were cultured in RPMI 1640 containing 10% fetal bovine serum (FBS), 2 mM glutamine and 1% penicillin/streptomycin (P/S). ES-2 and HEK293 cells were cultured in Dulbecco’s modified Eagle’s medium containing 10% fetal bovine serum (FBS) and 1% P/S. All cells were incubated at 37 °C in a humidified atmosphere containing 5% CO2. All experiments were performed with mycoplasma-free cells.

All the cells we used were purchased from ATCC with STR certification. We conducted a cell quality inspection in 2019 and found that they were free of HIV, HBV, HCV, mycoplasma, bacterial, yeast, and fungal contamination.

### Immunohistochemistry (IHC) staining

Immunohistochemical staining was performed as described. After treatment with diaminobenzidine and counterstaining with hematoxylin, all the sections were observed and photographed with a microscope (Axio Imager: Carl Zeiss). Scoring was conducted according to the ratio and intensity of positive-staining cells: 0–5% scored 0; 6–35% scored 1; 36–70% scored 2; more than 70% scored 3. The final scores were designated as low or high expression groups as follows: low expression: score 0–1; high expression: score 2–3. These scores were assigned independently and in a blinded manner by two senior pathologists. The primary antibodies used for FOXK2 detection are shown in Supplementary Table 2.

### siRNA transfection

Cells were plated at 60–70% confluence in 60 mm dishes. OVCAR8 and ES-2 cells were transfected with si- FOXK2 or with a nontargeted siRNA as a control. The sequences of the siRNAs used were as follows: si-FOXK2-1, sense (5′-3′): GCGAGUUCGAGUAUCUGAUTT, antisense (5′-3′): AUCAGAUACUCGAACUCGCTT; si-FOXK2-2, sense (5′-3′): CGGUGACCAUAGUACAACATT, antisense (5′-3′): UGUUGUACUAUGGUCACCGTT. SiRNA oligos were produced by Gene Pharma (Shanghai, China). Transfection steps were performed according to the manufacturer’s protocols using Lipofectamine® RNAiMAX (Thermo Fisher Scientific, Waltham, MA, USA).

### Plasmid transfection

The sequences of the short hairpin (sh)RNAs targeting FOXK2 were sh-1, 5′- GCGAGTTCGAGTATCTGATTT -3′ and sh-2, 5′- CGGTGACCATAGTACAACATT-3′. The shRNA-containing plasmids and a negative control plasmid were purchased from GenePharma (Shanghai, China). All these plasmids were packaged into virus particles using HEK 293T cells and the viral titers were determined. To establish stable FOXK2-knockdown cell lines, the target cells were infected with 1 × 10^8^ lentivirus-transducing units with 6 μg/mL polybrene (Sigma-Aldrich, St. Louis, MO, USA). The infected cells were then screened with 2.5 μg/mL puromycin after 72 h. The efficiency of the knockdown or overexpression was verified by western blotting. The plasmids containing FOXK2 (NM_004514.3, 4002) and PDK2(NM_002611.4, A3968) and a negative control plasmid were obtained from FulenGen Ltd., Co. (Guangzhou, China). The sequences of FOXK2-HA, FOXK2-Δ(54-128)-HA, FOXK2-Δ(129-171)-HA, FOXK2-Δ(300-371)-HA, and PDK2-Flag are shown in Supplementary Data.

### RNA isolation and quantitative real-time PCR

Total cellular RNA was extracted using TRIzol reagent (Takara). A PrimeScript RT-PCR kit (Takara) was used to perform the RT according to the protocol. SYBR Premix Ex Taq (Takara) on a 7500 real-time PCR system (Applied Biosystems) was used to determine the mRNA expression at the following cycling settings: one initial cycle at 95 °C for 10 s followed by 40 cycles of 5 s at 95 °C and 31 s at 60 °C. Data were normalized to 18S RNA expression and represented as the average of three repeated experiments. Prime sequences used for FOXK2, GLUT1, HK2, GPI1, PFK1, ALDOA, GAPDH, PGK2, PGAM2, ENO1, PKM2, LDHA, PDK2 and 18 s detection are shown in Supplementary Table 1.

### Western blotting

Total cellular protein was extracted using a total protein extraction buffer (Beyotime, China) according to the manufacturer’s instructions. Equal amounts of proteins were loaded onto 10% Tris-glycine sodium dodecyl sulfate‒polyacrylamide gel electrophoresis gels (Bio-Rad Laboratories, CA, USA). Then the separated proteins were transferred onto nitrocellulose membranes (Millipore, MA, USA). After blocking with 10% nonfat milk, the membranes were incubated with a primary antibody at 4 °C overnight. The membranes were further incubated with secondary antibody and protein signals were detected with the Odyssey imaging system (LI-COR Biosciences, Lincoln, NE). Primary antibodies against FOXK2, GLUT1, HK2, GPI1, PFKL, ALDOA, GAPDH, PGK1, PGAM1, ENO1, PKM2, LDHA, PDK2 and β-Actin used for detection are shown in Supplementary Table 2.

The phos-tag gels contained 20 μM Phos-tag (APExBIO, F4002) and 40 μM MnCl2. Before transferring onto nitrocellulose membrane, the phos-tag gel was immersed in transmembrane buffer containing 10 mM EDTA and washed to eliminate manganese ions.

### Cell viability assa*y*

The cells were plated in 96-well plates at a density of 3000 cells per well with 100 μl of complete culture medium and cultured for 2-5 days. Each group contained five wells. 10 μl Cell Counting Kit-8 (CCK-8, WST-8, Dojindo, Japan) solution was added to each well after 24 h, 48 h, 72 h and 96 h. CCK-8 was metabolized to produce a colorimetric dye that was read at 450 nm using a microplate reader.

### Cell migration

In the migration assay, 2.5 × 10^4^ cells were seeded into the upper chamber of the Transwell plate (Millipore, USA). Cells were allowed to migrate for 24 h at 37 °C. The migrated cells were then fixed and stained with 0.1% crystal violet, six randomly selected fields were photographed, and the cell numbers were counted.

### In vivo tumor xenograft model

Six-week-old female athymic nude (nu/nu) mice (SLAC, Shanghai, China) were randomly divided into four groups and injected subcutaneously in the right flank with the stable single cell clones of OVCAR8-sh and control cells at 5 × 10^6^ cells in 100 μl PBS medium for each nude mouse. We measured tumor volume once a week. After the mice were killed. The tumors were dissected and fixed with phosphate-buffered neutral formalin for standard histologic examination. The mice were manipulated and housed according to protocols approved by the East China Normal University Animal Care Commission.

### Seahorse analyses

The assays for extracellular acidification rate (ECAR) and oxygen consumption rate (OCR) in the cultured cells were performed with the Seahorse XF96 Flux Analyzer (Seahorse Bioscience, Agilent) according to the manufacturer’s instructions. Briefly, OVCAR8 and ES-2 cells were seeded in an XF96-well plate at a density of 1 × 10^4^ per well with the indicated treatments. The medium was replaced with the assay medium 1 h before the assay. For the glycolytic stress test (Seahorse Cat. #103020-100), 10 mM glucose, 1 μM oligomycin and 50 mM 2-deoxyglucose (2-DG) were injected into the wells. For the mitochondrial stress test (Seahorse Cat. #103015-100), 1 μM oligomycin, 1 μM FCCP, 0.5 μM rotenone and 0.5 μM antimycin A were added to the wells. The above experiments were performed in triplicate and repeated twice.

### Chromatin immunoprecipitation (ChIP) assay

ChIP assays were performed using a Pierce Agarose ChIP Kit (Thermo, 26156) according to the manufacturer’s instructions. Antibodies against FOXK2 (Abcam, ab5298, 4 μg per ChIP) were used for immunoprecipitation. Quantitative analysis of ChIP-derived DNA was performed by real-time qPCR analysis (primers in Supplementary Table S3). The assays were performed in triplicate.

### Plasmid construction and dual-luciferase reporter assay

HK2, GPI, GAPDH, PGK1, PKM2, LDH and PDK2 promoter-luciferase reporter plasmids containing the promoter region were constructed in the pGL4.10/pGL3B plasmid. Wild-type and mutant promoter luciferase constructs were verified by DNA sequencing. A dual luciferase reporter assay (Promega, WI, USA) was performed according to the manufacturer’s instructions.

### Co-immunoprecipitation (Co-IP) Assay

Cells were lysed in IP buffer (P0013, Beyotime) containing protease inhibitors (B14001, Selleck) and phosphatase inhibitors (B15001, Selleck). Protein A/G beads (Santa Cruz Biotechnology) were preincubated with antibody and IgG for 30–60 min on a spinning wheel. The bead-antibody complexes were then suspended with protein lysate. All Co-IP was performed overnight on a spinning wheel at 4 °C. The beads were washed 3 times with extraction buffer and were collected by centrifugation at 5000 rpm. The immunoprecipitants were subjected to western blotting.

### Immunofluorescence (IF)

Ovarian cancer cells were planted in 8-well chambers (Ibidi, Germany) for IF. We fixed cells with 4% polyformaldehyde (30 min), permeabilized them with 0.1% Triton X-100 (10 min) and blocked with 10% BSA (1 h) at room temperature. All cells were incubated with the primary antibodies at room temperature for 2 h and then labeled with Alexa Fluor-488-conjugated Alexa (1:400, Rabbit, Sigma, USA) and Fluor-594-conjugated secondary antibodies (1:400, Mouse, Sigma, USA) at room temperature. DAPI was used to stain the nucleus for 5 min (Sigma, USA). Confocal microscopy (LSM 510, METALaser scanning microscope, Zeiss) was used to acquire the images. The primary antibodies used for FOXK2 and PDK2 detection are shown in Supplementary Table 2.

### In vitro kinase assay

Immunoprecipitation (IP) of FOXK2 and PDK2 followed the same steps as the Co-IP assay to purify FOXK2 and PDK2 proteins. Equal aliquots of FOXK2 and PDK2 proteins were then subjected to kinase reactions. Kinase reactions were performed in kinase buffer (Cell Signaling Technology, #9802), for 30 min at 30 °C, with 200 µM ATP (Cell Signaling Technology, #9804). Then we used ADP-Glo™Kinase Assay (Promega, V6930) to measure kinase activity by quantifying the amount of ADP produced during a kinase reaction according to the manufacturer’s instructions. In addition, reactions were then stopped by the addition of 4X SDS‒PAGE buffer and loaded for separation on a 10% SDS‒PAGE gel.

### Liquid chromatography tandem mass spectrometry (LC-MS/MS)

Sample Preparation: Gel pieces were cut from SDS PAGE, destained in 100 mM NH4HCO3 with 30% Acetonitrile and washed with Milli-Q water for10 min until the gels were destained. The spots were then lyophilized in a vacuum centrifuge. The in-gel proteins were reduced with dithiothreitol (10 mM DTT/100 mM NH4HCO3) for 30 min at 56 °C, then alkylated with iodoacetamide (200 mM IAA/100 mM NH4HCO3) in the dark at room temperature for 20 min. Gel pieces were briefly rinsed with 100 mM NH4HCO3 and I, respectively. Gel pieces were digested overnight in 12.5 ng/μl trypsin in 25 mM NH4HCO3. The peptides were extracted three times with 60% I/0.1% TFA. The extracts were pooled and dried completely by a vacuum centrifuge.

LC-MS/MS: The peptide of each sample was desalted on C18 Cartridges (Empore™ SPE Cartridges, Sigma), then concentrated by vacuum centrifugation and reconstituted in 10 µl of 0.1% (v/v) Formic acid. MS experiments were performed on a Q Exactive HF mass spectrometer that was coupled to Easy nLC (Thermo Scientific). Peptide was first loaded onto a trap column (100 μm*20 mm, 5 μm, C18) with 0.1% formic acid, then separated by an analytical column (75 μm*100 mm, 3 μm, C18)) with a binary gradient of buffer A (0.1% Formic acid) and buffer B (84% acetonitrile and 0.1% Formic acid) at a flow rate of 300 nL/min over 60 min. The gradient was set as following: 2–7% buffer B from 0 min to 3 min, 7–35% buffer B from 3 min to 48 min, 35–90% buffer B from 48 min to 53 min, 90% buffer B kept till to 60 min. After peptide segment separation, DDA (data dependent collection) mass spectrometry analysis was performed using a Q-Exactive Plus mass spectrometer (Thermo Scientific).MS data was acquired using a data-dependent top20 method dynamically choosing the most abundant precursor ions from the survey scan (300–1800 m/z) for HCD fragmentation.The full MS scans were acquired at a resolution of 70,000 at m/z 200, and 17,500 at m/z 200 for MS/MS scan. The maximum injection time was set to for 50 ms for MS and 50 ms for MS/MS. Normalized collision energy was 27 and the isolation window was set to 2.0 Th.

Database search: The MS data were analyzed using MaxQuant software version 1.6.1.0. MS data were searched against the UniProtKB Human database (173,343 total entries, downloaded 09/2019). The trypsin was selected as digestion enzyme. The maximal two missed cleavage sites and the mass tolerance of 4.5 ppm for precursor ions and 20 ppm for fragment ions were defined for database search. Carbamidomethylation of cysteines was defined as fixed modification, while Oxidation (M), Acetyl (Protein N-term) and Phospho (STY) were set as variable modifications for database searching. The database search results were filtered and exported with <1% false discovery rate (FDR) at peptide level and protein level, respectively.

### Statistical analysis

Data are shown as means ± S.D. GraphPad Prism 10 was used to manipulate statistical analyses. Correlation of FOXK2 expression with categorical clinical variables in patients with OC was evaluated by χ2 test (SPSS 20.0 statistical software). After testing the homogeneity of variance, two-tailed Student’s *t* test was used to compare the results from different groups. Spearman rank correlation test was used to analyze the correlation between FOXK2 and glycolysis related genes. All experiments with cell lines were done in at least triplicates. *P* > 0.05 = NS, *P ≤ 0.05, **P ≤ 0.01, ***P ≤ 0.001, ****P ≤ 0.0001.

### Supplementary information

Supplementary materials

Figure S1

Figure S2

Figure S3

Figure S4

Table S1

Table S2

Table S3

Table S4

Table S5

## Data Availability

All data and materials were available from the corresponding authors on reasonable request.
