# Supplementary material for: Phosphorylation of FOXK2 at Thr13 and Ser30 by PDK2 sustains glycolysis through a positive feedback manner in ovarian cancer
Source: Oncogene. 2024 May 11;43(26):1985–99. doi: 10.1038/s41388-024-03052-x (PMC11196215; doi:10.1038/s41388-024-03052-x)
Supplement: Supplementary file 1 — Supplementary materials [file 41388_2024_3052_MOESM1_ESM.pdf]

Supplementary figures.

Fig. S1. FOXK2 directly regulate GAPDH in OC. ChIP assays followed by qPCR of the promoter region of PDK2 in OVCAR8 and ES-2 cells. Luciferase reporter assay was performed using OVCAR8 and ES-2 cells after transfecting the wild type plasmids and mutated plasmids (mutation site: orange). Data are presented as means ± SEM. *P < 0.05; ****P < 0.0001.

Fig. S2. Phosphorylation site of FOXK2 in PhosphoSitePlus.

Fig. S3. PDK2 phosphorylates FOXK2. The linear relationship between the luminescent signal and the amount of ATP in the kinase reaction buffer from 0–200μM using the Kinase-Glo®Assay (r2 = 0.999) and the amount of ATP remaining in solution following a kinase reaction. Equal aliquots of FOXK2 and PDK2 proteins were then subjected to Kinase reactions with 200 μM ATP.

Fig. S4. Phosphorylation mutant of FOXK2 at Thr13 and Ser30 failed to rescue the knockdown-induced suppression of proliferation and glycolysis. A-B: Relative cell viability OVCAR8 and ES-2 expressing si-FOXK2, and additional expression FOXK2 or mutant plasmids in OVCAR8 and ES-2 cells. **C-D:** Extracellular acid ratio (ECAR) upon overexpression of FOXK2 or mutant plasmids in OVCAR8 and ES-2 cells (ECAR, n=3). Glc: glucose; O: oligomycin; 2-DG： 2-deoxy-glucose.

Table S1. Primers used in the paper.

Table S2. Antibodies used in the paper.

Table S3. Primers used in the CHIP-PCR.

Table S4. The databases examining FOXK2 targets-glycolytic enzymes through ChIP Seq.

Table S5. Phosphorylation sites of FOXK2 in three independent mass spectrometry analyses.

The original sequence of HK2 promoter is as follows (1600bp), and the mutation site is marked with font.

Tagcttatcatctattgaggagaggaagaacatagcgaaaataaataggaaatcaactagggccaagtgatagtgacttgg
ggaactatttgaga[**taaac]**tggtcaaggaaagcctgatgaggtagaaggtggggacttgactctggaggtgggggcta
agactcgggaccagactctagattagagttccagatttaacacctagaagtcactgcccctttccatggcaatgactcaacaa
cccgttaccaaccttttctagaaatttctgtataacctgccccttaatttgcatgttaactaaaagtgggtagaaatatgagtgc
agagctgcctctgagctgctactctgggcacacggccttatggggtagccctgctctgcaaagaccagtgcctctgctcctg
atgtacactgccacttcaatataagctgctgtctaatgccacctgcttgcccttgaattttttttttttttgaaatggagtctctttct
gttgcccaggctggagtgcagtggcgcgatctcggctcactgcaagctccgcctcccgggttcacgccattctcctgcctc
agcctcccgagtagctgggactacaggagcccgccaccacgcctaatttttgtatttttttttttttagtagagatggggtttc
accgtgttagctaggatggtctcgatctcctgacctcgtgatccgtccacctcagcctcccaaagtgctgggattacaggtgt
gagccaccgcgcccggcatcccttgaattctttactgggtgaagccaaaaatcttcccaggctaagtccaaattttggggcc
tgcctgccctgcatcatgaggaggtatctgagtggaacgtcaatgaggaggaagaatgagttggagacaggcctggaga
agaatattctagatagaaggaaaaggaagagcaaagacccttgggtgagaaagagtttgtatttttgaggaaagcatgcta
gtgtgaatgccaagcagtattctgtgggaagatctcaggaggtgtctaagggcatggagataagtggtcagatgcacggtc
tgttttataggtggaattaactgcttgctgatggattgactggctgtgagggtgagtggcaagaaggaatcgaagacgagtta
gggtggatggcgatgccatttgctgagacaactgggaaagaaaaagatttgggaaaaaaagttgagttcagctttggacat
gttaagtgtgatatgctagtcacttcagtggagatgacaaatggcaagctggagaaataagcctgcaactccaggagagga
cctcgctgtagatttactatggtgagtcatcagcatgcatatgatataacagtcatggggctaggaagttagtttctcctcagg
gagtttgaaactgtaactagttcagagaagagggtggagggcagccccgaataccccagcatttaccaatagagc**[aaac
a]**gggactcaggagcctggggagtgaggttagccagaaaccctcagagtggagcactggtgctcttactgagagaggaa
ggtgtgtccagatggaggatgtgattaactgtcctcaacatccctgagagaaggagtaagaca

The original sequence of GPI promoter is as follows, and the mutation site is marked with font.

Ccagctggatgtcctctagttcaattcaattcgtctcacactgtctacctggagatactgtcagatcccactgattaagggctc
agtcccacaaaactgtgcccctcctttagaagccagttatgagttcaggcctccagtacttcgttgtttttttttttgtttttttgtttt
tttgtttttttgtgagtgggagtctcgctctgttgcccaagcttgagtgcagtggtgtgatcttggctcactgcaacctccgcccc
ccccccccccccgggttcaagggcttctcctgcctcagcctccagagtagctgggattacaggcacatgccaccacgc
ccagctaatttttggtagagacagcgtttcaccatgttggccaggctggtctcaaactactaacctcaggtgatctacccgtct
cagcctcccaaagtgctgggattacaagtgtgagccattgctcccggcccatataggatggaggtgagcaatttggcaagg
aagaaat**[taaaaa]**gcacataagaacagtatcaagaccattggaatcagatgacttcctgtccaagattcccagctgtgttt
gtgcaaataagacttgatagtagtggaaaacagggacaagaagagactgtctctttcttctcaggtagatccccaaaactga

aagccattttcaatcctgccacttgctcactaaagatttagggtttaggtccaaaccctaagtcttggattccttatcttctcaaac
atgctcttgtttgcttgatgcctggttcgagtggccaaggccgacattcatagacgttggttaatccctaatttcgccaaacaat
gacggcctaggcttttccggcttttgaaacaggaattcaagacactaaaacgtgtgttgatggcattttgggttggggcttttc
acaactgatgacactggaaaaataatccataggaaactaccttatgagaccctagatttcggtttag

The original sequence of PGK promoter is as follows, and the mutation site is marked with font.

Atatattttcccagcactgtgaataaaagtcagttgaatgagaaattgaaagtacctctgattggtcccttcctgcaaccaatc
aggctggtggcaggccaagttttcatttgtataggagtgtaactttgtaacttcacttcagcctctgattggtcactttctgcaac
caatcagactgaccatggaccactacttcatttgcatggggtatacaccaagtggctaatgggaaacctctaccgggtattta
gaccccagaaaattctgtaaccaagctcttgagctgcttgctcaggcctgctctcaccttgtggagtatacttttgaaaccgcc
tttgcaaaaatcataactgaggaaattatgacagtgaaagagatcagacctaacctaccctatcttccttctaacttctaaaatg
tctttgttcattcctgggtataggccaaactagccttgggaaagaatttatagtg**[taaac]**tctgagacaaaattgataacagc
cctttccccttcttgctgggaccagtctgcctttctttgtaggtgtaacaaattagctacaagattataaattacagtttaggggc
catgcagcctctggctgcaagaatctgaacctccccaaattgcttctgggaataacatcaccattgcaaatcctaagatcagt
gcttgagatattttgcagaccctgcattctcatgcagcagaggacaccacccagactgataatctggctcaactagctccac
gatcccacccaggaagaactcgcaagaactcacttcgaccccgtgatttaatcttcatcccgaccaactagcactccccact
ttccgaacccatacctgccaaattatccttaaaaactctgattccagaatgctcagggagactgatttgagtgataatgaaact
ctggtctcccgcacagcgctcagcgtgaattattctttcgccattgcaattcccgtcttcataaatc

The original sequence of GAPDH promoter is as follows, and the mutation site is marked with font.

Gtgaccacactggtgcctctgctgtcatgacaacagcacactacgtcagtagtgctccctgggcactgagctccctctttgc
ggggagaagacagtaatgaaaaatgacaagcatgaggcagaggggaagatcacgcttgggtggtgcaggagcatgga
ggtgctcttaatgctctcaatgagaaagggttaacggtcctggttgcaggaatagctgagtcagaggtggggcttcctccac
tcccccaccccacccctttcaccattagggaccttcttgccttgctcttgctactctgctctgggtggtcattgtgaaaagcccg
caccaaccatgccagtggcagccagacgaggacacagcctggctctgggtcccagcaggaaaggcaatcccagaaag
gcagggtcagggactggagtcctgtgggtgctttttaagcaaagattatcaccaggcaggc**[taaac]**ttagcaaccggct
tttagctagaagggcaggggggctggtgtcaggttatgctgggccagcaaagaggcccgggatccccctcccatgcacct
gctgatgggccaaggccaccccaccccacccccttccttacaagtgttcagcaccctcccatcccacactcacaaacctgg
ccctctgccctcctaccagaagaatggatcccctgtgggagggggcaggggacctgttcccaccgtgtgcccaagacctc
ttttcccactttttccctcttcttgactcaccctgccctcaatatcccccggcgcagccagtgaaagggagtccctggctcctg
gctcgcctgcacgtcccagggcggggagggacttccgccctcacgtcccgctcttcgccccaggctggatggaatgaaa
ggcacactgtctc

The original sequence of LDH promoter is as follows, and the mutation site is marked with font.

Cgggcgtggtggcgcctggctgtaatcccatcttctcaggaggctgaggcaggagattcgcttgaacccgggagg
cggaggttgcagtgagccgagatcagcccactgcactccagcctgggcgacagagcgactccgtttcaaaaaaaa
aaaaattaataattaaaaaaaaaatcgcagggcaagtgggcgcgcttgtagtcccagtcacttcggggagccgag
gtgggaggatcgcttgagccagaaggtcgaggctgcaggaagccatgatcacgccactgccctccagcctgggtg
acagagtgagaccctgtctcgaaaaataccaaaaaacaaa**[taaaca]**aacaaaaaaccaaaaccaaaaaaacaa
gccactgacagttcttgggtatggttgagactcgagatgagatgccagtggggtgggcagtagaaagtgcagaat
aaaatgtacatttgaactgagtcaccctgcaaggcctgagaggccaaggcttcactgtgagtgggagctggtagg

cttagcagcagagggaaaagcagcgtcgagttttggaggtcactcgacttaggtaagaacagactgactgactgc
taggcattttcttcctttcgttcaacaaatatttgtggagtgcctattacgtgccagaagctgttctggacactg
agaaacagggatgaagaagaaacagatccaagccttcctgagagtaacctccccaggtttcatggatgaggaaac
tgaaggtcgtcctgactcaggctcatggctccgaccccggcttctgtggttggagggcagcaccttacttagact
cccagcgcacgtggagcagtctgccggtcggttgtctggctgcgcgcgccacccgggcctctccagtgccccgcc
tgg

The original sequence of PFK promoter is as follows, and the mutation site is marked with font.

Gcagggaaaaggacagagggagctgacgattacacctaactttggatttggggcacccttctgttcccc
cacttcctgcttcgctgacactcacacccccaaaggcggtggcgcagaagctgaaatcagggctcagga
ctccatctacagccaggcccacgagcagggtctgttctgaaggctccaccagcagctgtgtcccaagcc
aactggcaagacaccgtcccacccgacagtggcttcctccccgtggcgtcatctcccttccaaagaggg
aggaatttggactattacccacgaacgatagcaggctaag[**aaaca**]aattatgattacgaaagattaa
agaaaaaaaaaaccacccacacctactggctgtacctttcctgcatgatttttgccaccgcatgggaag
cgggccaattgctatttgcctgggtgttttaaatatcaggagatcaggagatttgctcctgacataatt
ttttttcgaagtgcaacagaaaatagcacttttcacaacaattaactgttaactggctccaaagaaggt
ctgcttgttagacattagggaggaatagaaggcatcttctcagatgttggcctttgcggaggcaaggtc
agtggtgccgggaggtgactccctgcatttccgtatgtccgtttcttaagtcgcactattacttccctt
gagtaagggacgtaatattgagtaatatttattgagctgtgtgtgagagactgtttcaggctccggga[
aaaca]gcgaacaaaataaaaacaaattggctgctttcatagaccccacacactaataagcacataggg
agtcggtcaggcggtgacccgtgcggtggagagaaggcagccggcgggacgtggaagggtcgaggttaa
accgaggtagtggggaggcccccagtgaaagacgcgagtttccggggtgctccctctagccagca

The original sequence of PKM2 promoter is as follows, and the mutation site is marked with font.
Tgcgcaacattgtatctgtgaatgaaggcaagagttaacagctgtttaattgataactgctcgcatcat
tagttgctggctaacaactgggaaatcagaaaatgtcttgtagaaaaatgtaagaaaagttccaacaat
actgact[**taaaca**]cgacaaaggtgaaaacagaaatgctgactcctgcataggttatcggccctaatg
ttctgacttgatatttccagatgcccagctctgcgctaatatcaacaccgtctatttactttctactct
gaggcattcgctctgcaggattccagaccctactaaattattcacatggccccaaccggtccttccttg
ttccgcggtcctaacacaatgaatggtcctaagaggaaaacggcctcgcgctcccgctccaggcccact
tcgcagtccctagttctccctactgccgctccagtgccagagcccctccgaaggcggccaggacctcca
accacgcacaagtctgcagctctccccaactttccgttcagctcagtctccgagggtgcgccagagcag
acacccggaggagtggggagtggcagggcggggccgggagaatgctgccccggaacccataaatctggg
ccctgcccaggtaggccgggacagctggggtggcctgggccgagagccaagaaaagacaccccatctgg
cagcccaacttggcggcaacaggtggcccggcgcccgggggtctgggaggaaagtcgctccgggggcgg
gccccgttgccccgccgcgtccccattggtcatcaggtttcttaaaatgtgactctgaatctgtgtcct
tccgccgcagaatttagtcccaccgaaagggcaacctgcccgcgcgttccgccgccgccgccgcgcttc
ctcctgaaggtgactgcgcccgcggggacgcagggggcggggcccgggtcgcccggagccgggattggg
cagagggcggggcggcggagggattgcggcggcccgcagcgggataaccttgaggctgaggcagtggct
ccttgcacagcagctgcacgcgccgtggctccggatctcttcgtctttgcagcgtagcccgagtcggtc

agcgccggaggtgagcggtgcaggaggctacgccatcagtccccaccaagggccagtcgcccggctagt
gcggaatcccggcgcgccggccggccccgggcacgcaggcagggcggcgcaggatccagggcgtctggg

The original sequence of PDK2 promoter is as follows, and the mutation site is marked with font.

GAGACGGAAAGGGACAACCCCGGCTCCTCTCCAGGCGTGGAGTCTGTGGGGATGTGCTTCCAGAAAATG
CAGGGTTAAGCAGGAGCTGCAGAGTAGAATCAAATGACAATGACTCACTGCTGTCACTAACAGGCTCTT
TGTGGGGGCTGTAGGTGGGAGGCGATATGGCCGGCACCTTCGCACAAACCTCCCTTGCCCGTCAGCAAG
GTGTGTGGACCCCATACAGGGGGTGTGGCTTGGGTGCCCAGGACCTGCTAAAGCCACACGAGCTCTCCA
AACCCGTCAGCCGCTCCTTGCATGACCGAGCAGGACCGACCACTTACTGCGCATGTACTCGAGGCATCA
TCTTTTCTGTACACCCAGGCCCATGAGGTGCTTACTCCCATGAACCCCATTTTGCAAATGGGGACCCGA
GGGACTCGGTGGCCTCTTTAGGGCACAATCAGCAGCGGAACCCAAACCTGCCTGGCTCAGAGGCTCCGG
AGGCGGGACACCGCCACCTCTCCCCCAAGTGCAGCGAGCACCCTCCTCCCCAGCCTTTGCCTTGTTTAC
ACCCTGCTCCCCAGCAGCTGTGGATACGAGTGCACAGGG[**CACACC**]CTCGCACACACCCACAGGCATG
CACATGCCACATGCACACAATTACACACACTCCCCCGCTGGCTCAGCACCCCCTCTGCTCCCACACAGG
GCTGCAGGGCCCTCCTCCCACTCACTCACCCTCTCCTCCCGCCCCCACCGCCCATTATCAGCCCCACAC
TCTCAGCAAGCCTCCTGGCTTCTGCCTGCATCCACCCCTCCTCCTTCTCATTCCTGGGGCTCTGGACTG
GCTCTGGAGCCCTGGAAAGCAGGGTTTGGGTGGATCTCCACATTACCTTGGGCTGGGCTTGGGAGCTCC
GGATCTCACCAAGGGACTCCTGGGGGCCTTGATTACTGCAGAGGTCAGCAGGCCAGAGACCCCAGCCTC
CCTGCAGGTCCTGGGGAGGGGTGGGGGCAATGCCTCTCCCGCCTGTTGGTGCCTGTCTGTCACTGCACC
ACTGCTTCAGGCCTCCCTTATATAAGCCCAGTTCTCTCTCTGGCCTGCAAGGGGTGGGCAGCCCAGAGG
CCAGCGCCACTCGTCCCCAATCTCCCTTCTTAGGATCAAGAACCGGCCCAAGGTTGACATTTGGTGCAC
CAGGCCCCTGAGGGGCTTC

Plasmid sequence

FOXK2-HA （***Green marked the FOXK2-HA tag sequence, underlined is the restriction site)**

ATGCCCAGTACATGACCTTATGGGACTTTCCTACTTGGCAGTACATCTACG
TATTAGTCATCGCTATTACCATGGTGATGCGGTTTTGGCAGTACATCAATG
GGCGTGGATAGCGGTTTGACTCACGGGGATTTCCAAGTCTCCACCCCATT
GACGTCAATGGGAGTTTGTTTTGGCACCAAAATCAACGGGACTTTCCAAA
ATGTCGTAACAACTCCGCCCCATTGACGCAAATGGGCGGTAGGCGTGTAC
GGTGGGAGGTCTATATAAGCAGAGCTCTCTGGCTAACTAGAGAACCCACT
GCTTACTGGCTTATCGAAATTAATACGACTCACTATAGGGAGACCCAAGC
TG<u>**GCTAGC**</u>GCCACCATGTACCCATACGATGTTCCAGATTACGCTGCGGCG
GCCGCGGCGGCGCTCTCGGGCGCGGGCACGCCACCCGCGGGCGGCGGGG
CCGGGGGCGGCGGGGCCGGGGGCGGCGGGTCCCCGCCGGGCGGCTGGGC

CGTGGCGCGCCTGGAGGGCCGCGAGTTCGAGTATCTGATGAAGAAGCGCT
CGGTGACCATCGGCCGCAACTCGTCGCAGGGCTCGGTGGACGTGAGCATG
GGCCACTCGAGCTTCATCTCCCGGCGCCACCTCGAGATCTTCACGCCCCCG
GGCGGCGGCGGCCATGGCGGGGCCGCTCCGGAGCTGCCGCCCGCGCAGC
CCAGGCCCGACGCCGGCGGCGACTTCTACCTGCGCTGCTTGGGCAAGAAC
GGGGTATTCGTGGACGGCGTGTTCCAGAGGCGCGGGGCGCCGCCGCTGCA
GCTGCCGCGCGTGTGCACATTCAGGTTCCCGAGCACAAACATCAAGATAA
CGTTCACTGCCCTGTCCAGCGAGAAGAGAGAGAAGCAGGAGGCGTCTGA
GTCTCCAGTGAAGGCCGTACAGCCACACATCTCGCCCCTGACCATCAACA
TTCCAGACACCATGGCCCACCTCATCAGCCCTCTGCCCTCCCCCACGGGAA
CCATCAGCGCTGCAAACTCCTGCCCCTCCAGCCCCCGGGGAGCGGGGTCT
TCAGGGTACAAGGTGGGCCGAGTGATGCCATCTGACCTCAATTTAATGGC
TGACAACTCACAGCCTGAAAATGAAAAGGAAGCTTCAGGTGGAGACAGC
CCGAAGGATGATTCAAAGCCGCCTTACTCCTACGCGCAGCTGATAGTTCA
GGCGATTACGATGGCTCCCGACAAACAGCTCACCCTGAACGGGATTTATA
CACACATCACTAAAAATTATCCCTACTACAGGACTGCGGACAAGGGCTGG
CAGAATTCAATTCGCCACAATCTCTCTCTGAATCGTTATTTCATCAAAGTG
CCGCGTTCCCAGGAAGAACCAGGCAAAGGCTCGTTCTGGAGGATAGACCC
AGCCTCTGAAAGCAAATTAATAGAACAGGCTTTTAGGAAACGACGGCCTA
GGGGCGTGCCCTGCTTTAGAACCCCTCTGGGACCGCTCTCTTCTAGGAGTG
CCCCAGCCTCTCCCAATCACGCGGGAGTGCTGTCTGCTCACTCTAGTGGCG
CCCAGACCCCTGAGAGCCTGTCGAGGGAAGGTTCGCCGGCCCCCCTGGAG
CCTGAGCCTGGCGCTGCACAGCCCAAACTCGCTGTCATCCAGGAAGCCCG
GTTTGCCCAGAGCGCCCCAGGGTCACCTCTGTCCAGTCAGCCAGTCTTAAT
CACCGTCCAGCGGCAGCTACCACAGGCCATCAAGCCTGTCACCTACACTG
TGGCCACCCCAGTGACCACCTCGACCTCCCAGCCACCCGTCGTGCAGACG
GTTCACGTCGTCCACCAGATCCCAGCGGTGTCGGTCACCAGTGTGGCCGG
ACTGGCCCCAGCGAACACGTACACTGTCTCTGGACAAGCTGTGGTCACCC
CGGCAGCCGTGCTGGCCCCTCCTAAGGCAGAGGCCCAGGAGAATGGAGA
CCACAGGGAAGTCAAAGTGAAAGTAGAGCCTATTCCCGCCATTGGCCACG
CCACGCTCGGCACTGCCAGCCGGATCATTCAGACGGCACAGACCACCCCG
GTCCAGACGGTGACCATAGTACAACAGGCACCTCTAGGTCAACACCAGCT
ACCAATAAAAACTGTAACACAAAACGGCACTCACGTGGCATCAGTCCCCA
CTGCGGTCCACGGCCAGGTGAACAATGCCGCGGCGAGTCCTTTGCACATG
TTGGCAACACACGCATCCGCATCGGCCTCCCTGCCCACAAAGCGCCACAA
CGGTGACCAGCCGGAGCAGCCGGAGCTGAAGCGGATCAAGACAGAAGAC
GGCGAGGGCATCGTCATTGCCCTGAGCGTGGACACGCCACCGGCAGCCGT
AAGGGAAAAGGGTGTCCAGAACTAG**GGATCC**ACTAGTCCAGTGTGGTGG
AATTCTGCAGATATCCAGCACAGTGGCGGCCGCTCGAGTCTAGAGGGCCC
GTTTAAACCCGCTGATCAGCCTCGACTGTGCCTTCTAGTTGCCAGCCATCT
GTTGTTTGCCCCTCCCCCGTGCCTTCCTTGACCCTGGAAGGTGCCACTCCC
ACTGTCCTTTCCTAATAAAATGAGGAAATTGCATCGCATTGTCTGAGTAGG
TGTCATTCTATTCTGGGGGGTGGGGTGGGGCAGGACAGCAAGGGGGAGG
ATTGGGAAGACAATAGCA

FOXK2-HA（△54-128 aa）
ATGTACCCATACGATGTTCCAGATTACGCTGCGGCGGCCGCGGCGGCGCT
CTCGGGCGCGGGCACGCCACCCGCGGGCGGCGGGGCCGGGGGCGGCGGG
GCCGGGGGCGGCGGGTCCCCGCCGGGCGGCTGGGCCGTGGCGCGCCTGG
AGGGCCGCGAGTTCGAGTATCTGATGAAGAAGCGCTCGAGGCGCGGGGC
GCCGCCGCTGCAGCTGCCGCGCGTGTGCACATTCAGGTTCCCGAGCACAA
ACATCAAGATAACGTTCACTGCCCTGTCCAGCGAGAAGAGAGAGAAGCA
GGAGGCGTCTGAGTCTCCAGTGAAGGCCGTACAGCCACACATCTCGCCCC
TGACCATCAACATTCCAGACACCATGGCCCACCTCATCAGCCCTCTGCCCT
CCCCCACGGGAACCATCAGCGCTGCAAACTCCTGCCCCTCCAGCCCCCGG
GGAGCGGGGTCTTCAGGGTACAAGGTGGGCCGAGTGATGCCATCTGACCT
CAATTTAATGGCTGACAACTCACAGCCTGAAAATGAAAAGGAAGCTTCAG
GTGGAGACAGCCCGAAGGATGATTCAAAGCCGCCTTACTCCTACGCGCAG
CTGATAGTTCAGGCGATTACGATGGCTCCCGACAAACAGCTCACCCTGAA
CGGGATTTATACACACATCACTAAAAATTATCCCTACTACAGGACTGCGG
ACAAGGGCTGGCAGAATTCAATTCGCCACAATCTCTCTCTGAATCGTTATT
TCATCAAAGTGCCGCGTTCCCAGGAAGAACCAGGCAAAGGCTCGTTCTGG
AGGATAGACCCAGCCTCTGAAAGCAAATTAATAGAACAGGCTTTTAGGAA
ACGACGGCCTAGGGGCGTGCCCTGCTTTAGAACCCCTCTGGGACCGCTCT
CTTCTAGGAGTGCCCCAGCCTCTCCCAATCACGCGGGAGTGCTGTCTGCTC
ACTCTAGTGGCGCCCAGACCCCTGAGAGCCTGTCGAGGGAAGGTTCGCCG
GCCCCCCTGGAGCCTGAGCCTGGCGCTGCACAGCCCAAACTCGCTGTCAT
CCAGGAAGCCCGGTTTGCCCAGAGCGCCCCAGGGTCACCTCTGTCCAGTC
AGCCAGTCTTAATCACCGTCCAGCGGCAGCTACCACAGGCCATCAAGCCT
GTCACCTACACTGTGGCCACCCCAGTGACCACCTCGACCTCCCAGCCACC
CGTCGTGCAGACGGTTCACGTCGTCCACCAGATCCCAGCGGTGTCGGTCA
CCAGTGTGGCCGGACTGGCCCCAGCGAACACGTACACTGTCTCTGGACAA
GCTGTGGTCACCCCGGCAGCCGTGCTGGCCCCTCCTAAGGCAGAGGCCCA
GGAGAATGGAGACCACAGGGAAGTCAAAGTGAAAGTAGAGCCTATTCCC
GCCATTGGCCACGCCACGCTCGGCACTGCCAGCCGGATCATTCAGACGGC
ACAGACCACCCCGGTCCAGACGGTGACCATAGTACAACAGGCACCTCTAG
GTCAACACCAGCTACCAATAAAAACTGTAACACAAAACGGCACTCACGTG
GCATCAGTCCCCACTGCGGTCCACGGCCAGGTGAACAATGCCGCGGCGAG
TCCTTTGCACATGTTGGCAACACACGCATCCGCATCGGCCTCCCTGCCCAC
AAAGCGCCACAACGGTGACCAGCCGGAGCAGCCGGAGCTGAAGCGGATC
AAGACAGAAGACGGCG

FOXK2-HA（△129-171 aa）
ATGTACCCATACGATGTTCCAGATTACGCTGCGGCGGCCGCGGCGGCGCT
CTCGGGCGCGGGCACGCCACCCGCGGGCGGCGGGGCCGGGGGCGGCGGG
GCCGGGGGCGGCGGGTCCCCGCCGGGCGGCTGGGCCGTGGCGCGCCTGG
AGGGCCGCGAGTTCGAGTATCTGATGAAGAAGCGCTCGGTGACCATCGGC
CGCAACTCGTCGCAGGGCTCGGTGGACGTGAGCATGGGCCACTCGAGCTT

CATCTCCCGGCGCCACCTCGAGATCTTCACGCCCCCGGGCGGCGGCGGCC
ATGGCGGGGCCGCTCCGGAGCTGCCGCCCGCGCAGCCCAGGCCCGACGCC
GGCGGCGACTTCTACCTGCGCTGCTTGGGCAAGAACGGGGTATTCGTGGA
CGGCGTGTTCCAGGTGAAGGCCGTACAGCCACACATCTCGCCCCTGACCA
TCAACATTCCAGACACCATGGCCCACCTCATCAGCCCTCTGCCCTCCCCCA
CGGGAACCATCAGCGCTGCAAACTCCTGCCCCTCCAGCCCCCGGGGAGCG
GGGTCTTCAGGGTACAAGGTGGGCCGAGTGATGCCATCTGACCTCAATTT
AATGGCTGACAACTCACAGCCTGAAAATGAAAAGGAAGCTTCAGGTGGA
GACAGCCCGAAGGATGATTCAAAGCCGCCTTACTCCTACGCGCAGCTGAT
AGTTCAGGCGATTACGATGGCTCCCGACAAACAGCTCACCCTGAACGGGA
TTTATACACACATCACTAAAAATTATCCCTACTACAGGACTGCGGACAAG
GGCTGGCAGAATTCAATTCGCCACAATCTCTCTCTGAATCGTTATTTCATC
AAAGTGCCGCGTTCCCAGGAAGAACCAGGCAAAGGCTCGTTCTGGAGGAT
AGACCCAGCCTCTGAAAGCAAATTAATAGAACAGGCTTTTAGGAAACGAC
GGCCTAGGGGCGTGCCCTGCTTTAGAACCCCTCTGGGACCGCTCTCTTCTA
GGAGTGCCCCAGCCTCTCCCAATCACGCGGGAGTGCTGTCTGCTCACTCTA
GTGGCGCCCAGACCCCTGAGAGCCTGTCGAGGGAAGGTTCGCCGGCCCCC
CTGGAGCCTGAGCCTGGCGCTGCACAGCCCAAACTCGCTGTCATCCAGGA
AGCCCGGTTTGCCCAGAGCGCCCCAGGGTCACCTCTGTCCAGTCAGCCAG
TCTTAATCACCGTCCAGCGGCAGCTACCACAGGCCATCAAGCCTGTCACC
TACACTGTGGCCACCCCAGTGACCACCTCGACCTCCCAGCCACCCGTCGT
GCAGACGGTTCACGTCGTCCACCAGATCCCAGCGGTGTCGGTCACCAGTG
TGGCCGGACTGGCCCCAGCGAACACGTACACTGTCTCTGGACAAGCTGTG
GTCACCCCGGCAGCCGTGCTGGCCCCTCCTAAGGCAGAGGCCCAGGAGAA
TGGAGACCACAGGGAAGTCAAAGTGAAAGTAGAGCCTATTCCCGCCATTG
GCCACGCCACGCTCGGCACTGCCAGCCGGATCATTCAGACGGCACAGACC
ACCCCGGTCCAGACGGTGACCATAGTACAACAGGCACCTCTAGGTCAACA
CCAGCTACCAATAAAAACTGTAACACAAAACGGCACTCACGTGGCATCAG
TCCCCACTGCGGTCCACGGCCAGGTGAACAATGCCGCGGCGAGTCCTTTG
CACATGTTGGCAACACACGCATCCGCATCGGCCTCCCTGCCCACAAAGCG
CCACAACGGTGACCAGCCGGAGCAGCCGGAGCTGAAGCGGATCAAGACA
GAAGACGGCGAGGGCATCGTCATTGCCCTGAGCGTGGACACGCCACCGGC
AGCCGTAAGGGAAAAGGGTGTCCAGAACTAG

FOXK2-HA（△300-353 aa）

ATGTACCCATACGATGTTCCAGATTACGCTGCGGCGGCCGCGGCGGCGCT
CTCGGGCGCGGGCACGCCACCCGCGGGCGGCGGGGCCGGGGGCGGCGGG
GCCGGGGGCGGCGGGTCCCCGCCGGGCGGCTGGGCCGTGGCGCGCCTGG
AGGGCCGCGAGTTCGAGTATCTGATGAAGAAGCGCTCGGTGACCATCGGC
CGCAACTCGTCGCAGGGCTCGGTGGACGTGAGCATGGGCCACTCGAGCTT
CATCTCCCGGCGCCACCTCGAGATCTTCACGCCCCCGGGCGGCGGCGGCC
ATGGCGGGGCCGCTCCGGAGCTGCCGCCCGCGCAGCCCAGGCCCGACGCC
GGCGGCGACTTCTACCTGCGCTGCTTGGGCAAGAACGGGGTATTCGTGGA
CGGCGTGTTCCAGAGGCGCGGGGCGCCGCCGCTGCAGCTGCCGCGCGTGT
GCACATTCAGGTTCCCGAGCACAAACATCAAGATAACGTTCACTGCCCTG

TCCAGCGAGAAGAGAGAGAAGCAGGAGGCGTCTGAGTCTCCAGTGAAGG
CCGTACAGCCACACATCTCGCCCCTGACCATCAACATTCCAGACACCATG
GCCCACCTCATCAGCCCTCTGCCCTCCCCCACGGGAACCATCAGCGCTGC
AAACTCCTGCCCCTCCAGCCCCCGGGGAGCGGGGTCTTCAGGGTACAAGG
TGGGCCGAGTGATGCCATCTGACCTCAATTTAATGGCTGACAACTCACAG
CCTGAAAATGAAAAGGAAGCTTCAGGTGGAGACAGCCCGAAGGATGATT
CAAAGCCGCCTTACTCCTACGCGCAGCTGATAGTTCAGGCGATTACGATG
GCTCCCGACAAACAGCTCACCCTGAACGGGATTTATACACACATCACTAA
AAATTATCCCTACTACAGGACTGCGGACGGCGTGCCCTGCTTTAGAACCC
CTCTGGGACCGCTCTCTTCTAGGAGTGCCCCAGCCTCTCCCAATCACGCGG
GAGTGCTGTCTGCTCACTCTAGTGGCGCCCAGACCCCTGAGAGCCTGTCG
AGGGAAGGTTCGCCGGCCCCCCTGGAGCCTGAGCCTGGCGCTGCACAGCC
CAAACTCGCTGTCATCCAGGAAGCCCGGTTTGCCCAGAGCGCCCCAGGGT
CACCTCTGTCCAGTCAGCCAGTCTTAATCACCGTCCAGCGGCAGCTACCAC
AGGCCATCAAGCCTGTCACCTACACTGTGGCCACCCCAGTGACCACCTCG
ACCTCCCAGCCACCCGTCGTGCAGACGGTTCACGTCGTCCACCAGATCCC
AGCGGTGTCGGTCACCAGTGTGGCCGGACTGGCCCCAGCGAACACGTACA
CTGTCTCTGGACAAGCTGTGGTCACCCCGGCAGCCGTGCTGGCCCCTCCTA
AGGCAGAGGCCCAGGAGAATGGAGACCACAGGGAAGTCAAAGTGAAAGT
AGAGCCTATTCCCGCCATTGGCCACGCCACGCTCGGCACTGCCAGCCGGA
TCATTCAGACGGCACAGACCACCCCGGTCCAGACGGTGACCATAGTACAA
CAGGCACCTCTAGGTCAACACCAGCTACCAATAAAAACTGTAACACAAAA
CGGCACTCACGTGGCATCAGTCCCCACTGCGGTCCACGGCCAGGTGAACA
ATGCCGCGGCGAGTCCTTTGCACATGTTGGCAACACACGCATCCGCATCG
GCCTCCCTGCCCACAAAGCGCCACAACGGTGACCAGCCGGAGCAGCCGG
AGCTGAAGCGGATCAAGACAGAAGACGGCGAGGGCATCGTCATTGCCCT
GAGCGTGGACACGCCACCGGCAGCCGTAAGGGAAAAGGGTGTCCAGAAC
TAG

PDK2-FLAG（ID: 5162）Green marked the FLAG tag
ATGGAT TAC AAG GAT GAC GAC
GATAAGCGCTGGGTGTGGGCGCTGCTGAAGAATGCGTCCCTGGCAGGGGC
GCCCAAGTACATAGAGCACTTCAGCAAGTTCTCCCCGTCCCCGCTGTCCAT
GAAGCAGTTTCTGGACTTCGGATCCAGCAATGCCTGTGAGAAAACCTCCT
TCACCTTCCTCAGGCAGGAGCTGCCTGTGCGCCTGGCCAACATCATGAAA
GAGATCAACCTGCTTCCCGACCGAGTGCTGAGCACACCCTCCGTGCAGCT
GGTGCAGAGCTGGTATGTCCAGAGCCTCCTGGACATCATGGAGTTCCTGG
ACAAGGATCCCGAGGACCATCGCACCCTGAGCCAGTTCACTGACGCCCTG
GTCACCATCCGGAACCGGCACAACGACGTGGTGCCCACCATGGCACAAGG
CGTGCTTGAGTACAAGGACACCTACGGCGATGACCCCGTCTCCAACCAGA
ACATCCAGTACTTCCTGGACCGCTTCTACCTCAGCCGCATCTCCATCCGCA
TGCTCATCAACCAGCACACCCTCATCTTTGATGGCAGCACCAACCCAGCC
CATCCCAAACACATCGGCAGCATCGACCCCAACTGCAACGTCTCTGAGGT
GGTCAAAGATGCCTACGACATGGCTAAGCTCCTGTGTGACAAGTATTACA

TGGCCTCACCTGACCTGGAGATCCAGGAGATCAATGCAGCCAACTCCAAA
CAGCCGATTCACATGGTCTACGTCCCCTCCCACCTCTACCACATGCTCTTT
GAGCTCTTCAAGAATGCCATGAGGGCGACTGTGGAAAGCCATGAGTCCAG
CCTCATTCTCCCACCCATCAAGGTCATGGTGGCCTTGGGTGAGGAAGATCT
GTCCATCAAGATGAGTGACCGAGGTGGGGGTGTTCCCTTGAGGAAGATTG
AGCGACTCTTCAGCTACATGTACTCCACAGCACCCACCCCCCAGCCTGGC
ACCGGGGGAACGCCGCTGGCTGGCTTTGGTTATGGGCTCCCCATTTCCCGC
CTCTACGCCAAGTACTTCCAGGGAGACCTGCAGCTCTTCTCCATGGAAGG
CTTTGGGACCGATGCTGTCATCTATCTCAAGGCCCTGTCCACGGACTCGGT
GGAGCGCCTGCCTGTCTACAACAAGTCAGCCTGGCGCCACTACCAGACCA
TCCAGGAGGCCGGCGACTGGTGTGTGCCCAGCACGGAGCCCAAGAACAC
GTCCACGTACCGCGTCAGCTAA
