# Supplementary figures and images for: Phosphorylation of FOXK2 at Thr13 and Ser30 by PDK2 sustains glycolysis through a positive feedback manner in ovarian cancer
Source: Oncogene. 2024 May 11;43(26):1985–99. doi: 10.1038/s41388-024-03052-x (PMC11196215; doi:10.1038/s41388-024-03052-x)

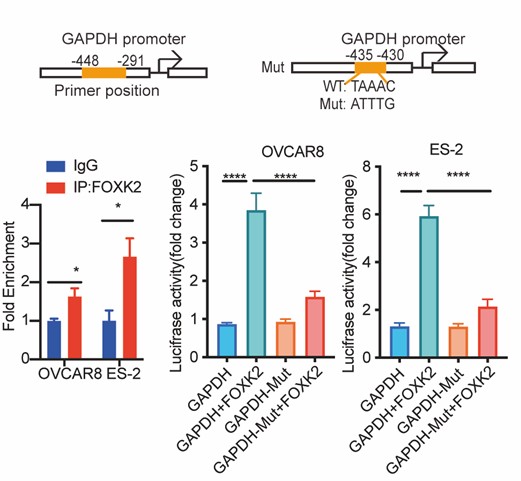

Supplement: Supplementary file 2 — Figure S1 [file 41388_2024_3052_MOESM2_ESM.jpg]

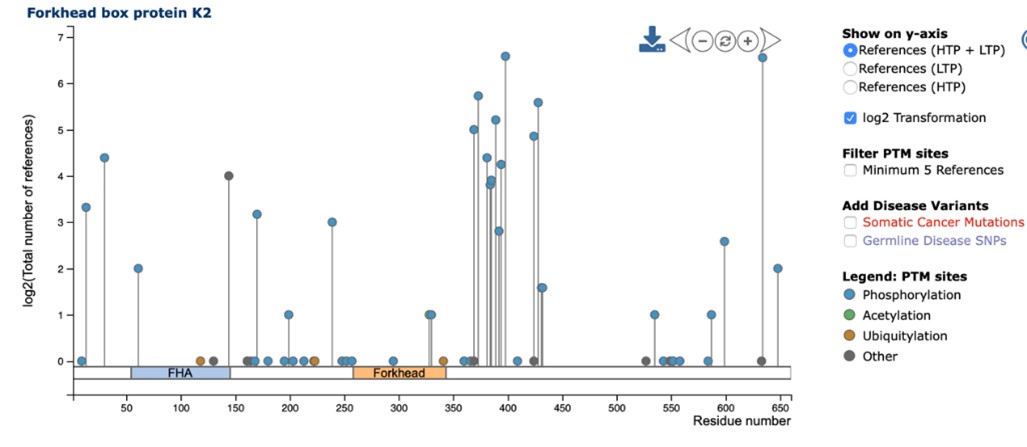

Supplement: Supplementary file 3 — Figure S2 [file 41388_2024_3052_MOESM3_ESM.jpg]

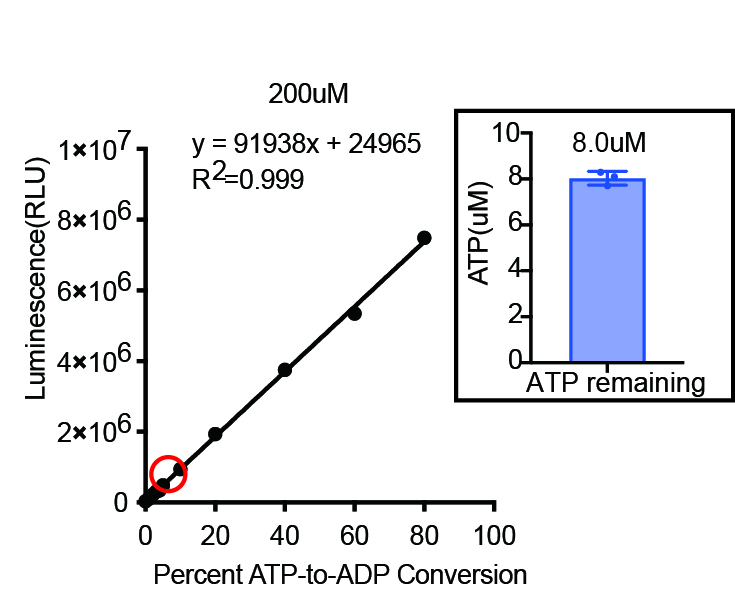

Supplement: Supplementary file 4 — Figure S3 [file 41388_2024_3052_MOESM4_ESM.jpg]

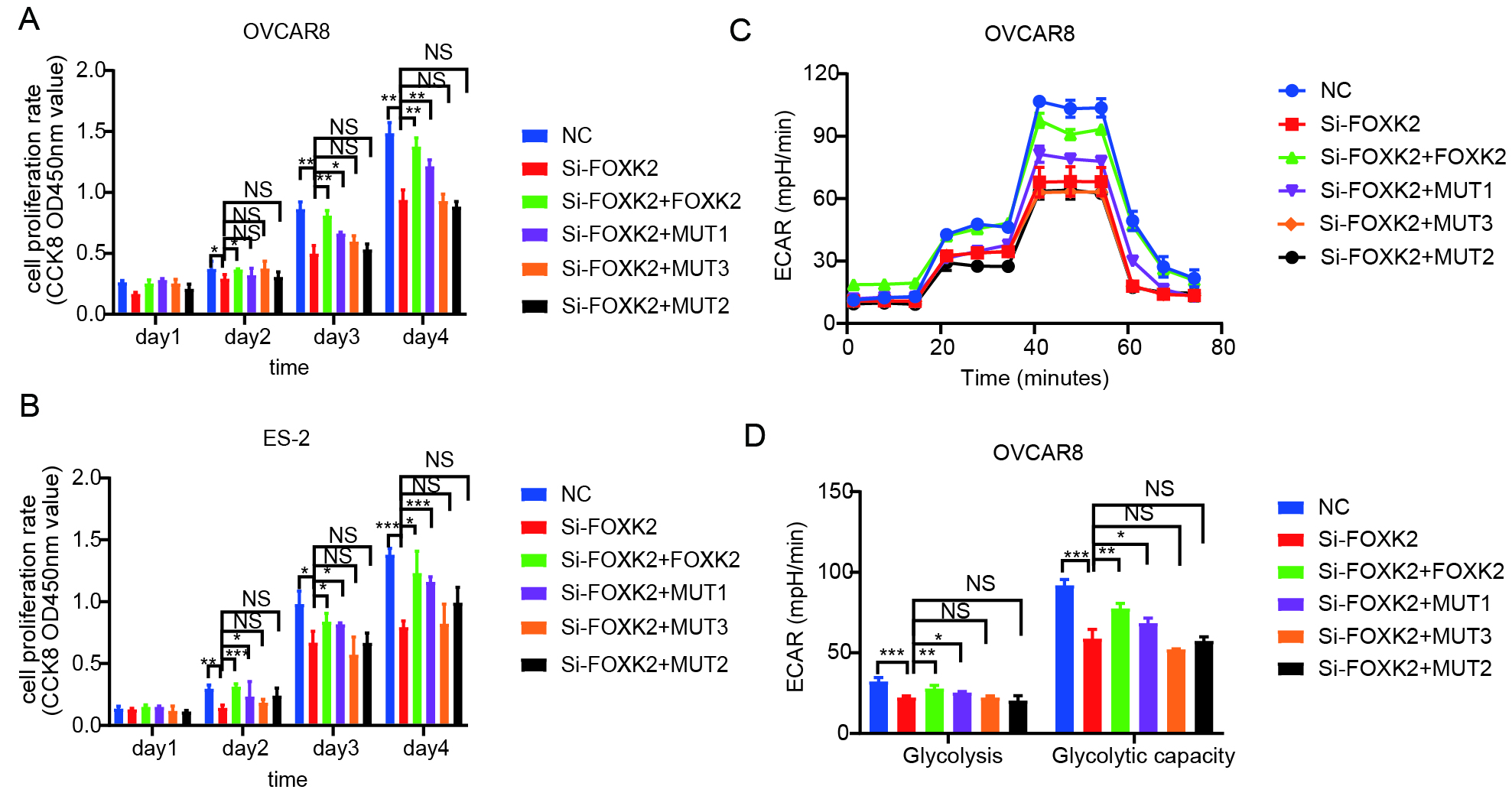

Supplement: Supplementary file 5 — Figure S4 [file 41388_2024_3052_MOESM5_ESM.jpg]
