# Supplementary material for: Phosphorylation of FOXK2 at Thr13 and Ser30 by PDK2 sustains glycolysis through a positive feedback manner in ovarian cancer
Source: Oncogene. 2024 May 11;43(26):1985–99. doi: 10.1038/s41388-024-03052-x (PMC11196215; doi:10.1038/s41388-024-03052-x)
Supplement: Supplementary file 6 — Table S1 [file 41388_2024_3052_MOESM6_ESM.docx]

Table S1. Primers used in the paper.

| Gene | Primer |
| --- | --- |
| FOXK2-F | AAGAACGGGGTATTCGTGGAC |
| FOXK2-R | CTCGGGAACCTGAATGTGC |
| GLUT1-F | ATTGGCTCCGGTATCGTCAAC |
| GLUT1-R | GCTCAGATAGGACATCCAGGGTA |
| HK2-F | TTGACCAGGAGATTGACATGGG |
| HK2-R | TTGACCAGGAGATTGACATGGG |
| GPI1-F | CAAGGACCGCTTCAACCACTT |
| GPI1-R | CCAGGATGGGTGTGTTTGACC |
| PFK1-F | AGCTGCCTACAACCTGGTGA |
| PFK1-R | TCCACTCAGAACGGAAGGTGT |
| ALDOA-F | CAGGGACAAATGGCGAGACTA |
| ALDOA-R | CAGGGACAAATGGCGAGACTA |
| GAPDH-F | GGAGCGAGATCCCTCCAAAAT |
| GAPDH-R | GGCTGTTGTCATACTTCTCATGG |
| PGK2-F | AAACTGGATGTTAGAGGGAAGCG |
| PGK2-R | GCGCCTATCTCTTTCCATCAGA |
| PGAM2-F | AGAAGCACCCCTACTACAACTC |
| PGAM2-R | TCTGGGGAACAATCTCCTCGT |
| ENO1-F | GCCGTGAACGAGAAGTCCTG |
| ENO1-R | GCCGTGAACGAGAAGTCCTG |
| PKM2-F | CAGAGGCTGCCATCTACCAC |
| PKM2-R | CCAGACTTGGTGAGGACGAT |
| LDHA-F | ATGGCAACTCTAAAGGATCAGC |
| LDHA-R | CCAACCCCAACAACTGTAATCT |
| PDK2-F | ATGGCAGTCCTCCTCTCTGAA |
| PDK2-F | CACCCACCCTCTTCCTAACA |
| RPS18-F | ATCACCATTATGCAGAATCCACG |
| RPS18-R | GACCTGGCTGTATTTTCCATCC |
