# Supplementary material for: Phosphorylation of FOXK2 at Thr13 and Ser30 by PDK2 sustains glycolysis through a positive feedback manner in ovarian cancer
Source: Oncogene. 2024 May 11;43(26):1985–99. doi: 10.1038/s41388-024-03052-x (PMC11196215; doi:10.1038/s41388-024-03052-x)
Supplement: Supplementary file 7 — Table S2 [file 41388_2024_3052_MOESM7_ESM.docx]

**Table S2.**  Antibodies used in the paper.

| Gene | Catalog Number | Applications |
| --- | --- | --- |
| FOXK2 | Abcam, ab5298, Goat | IF: 1:100; ChIP:4ug per ChIP. |
| FOXK2 | BETHYL, A301-729A, Rabbit | WB:1/1000, IHC: 1/100, IP: 1/10 |
| PDK2 | Abcam, ab68164 | WB:1/1000, IP: 1/20 |
| GLUT1 | Abcam, ab115730 | WB:1/2000 |
| HK2 | Abcam, ab209847 | WB:1/1000 |
| GPI | Abcam, ab167394 | WB: 1/1000 |
| PFKL | Abcam, ab181064 | WB: 1/1000 |
| ALDOA | Abcam, ab169544 | WB: 1/1000 |
| GAPDH | Abcam, ab181602 | WB: 1/10000 |
| PGK1 | Abcam, ab199438 | WB: 1/2000 |
| PGAM1 | Abcam, ab129191 | WB: 1/1000 |
| ENO1 | Abcam, ab227978 | WB: 1/1000 |
| PKM2 | Abcam, ab150377 | WB: 1/5000 |
| LDHA | Abcam, ab10156 | WB:1/2000 |
| PDK2 | Santa cruz, sc-100534, Mouse | WB:1/200 |
| p-Thr | Santa cruz, sc-5267, Mouse | WB:1/500 |
| p-Ser | Santa cruz, sc-81514, Mouse | WB:1/500 |
| P-Tyr | Abcam, ab179530, Rabbit | WB:1/1000 |
| β-ACTIN | Proteintech, 66009-1-Ig, Mouse | WB:1/10000 |
| HA tag | Abcam, ab236632 | WB:1/1000, IP: 1/30 |
| Flag tag | Abcam, ab205606 | WB:1/1000, IP: 1/30 |
