# Supplementary material for: Phosphorylation of FOXK2 at Thr13 and Ser30 by PDK2 sustains glycolysis through a positive feedback manner in ovarian cancer
Source: Oncogene. 2024 May 11;43(26):1985–99. doi: 10.1038/s41388-024-03052-x (PMC11196215; doi:10.1038/s41388-024-03052-x)
Supplement: Supplementary file 8 — Table S3 [file 41388_2024_3052_MOESM8_ESM.docx]

Table S3. Primers used in the CHIP-PCR.

| GAPDH-promoter-F: | ATCACCAGGCAGGCTAAACTT |
| --- | --- |
| GAPDH-promoter-R: | CACTTGTAAGGAAGGGGGTGG |
| PFK-promoter-F: | TTTCAGGCTCCGGGAAAACA |
| PFK-promoter-R: | TGACCGACTCCCTATGTGCT |
| LDH promoter-F: | CAGCGTCGAGTTTTGGAGGT |
| LDH promoter-R: | AACCTGGGGAGGTTACTCTCA |
| PDK2-promoter-F: | CCAGCAGCTGTGGATACGAG |
| PDK2-promoter-R: | GAGAAGGAGGAGGGGTGGAT |
| HK2 promoter-F: | GTGGAACGTCAATGAGGAGGA |
| HK2 promoter-R: | CACCCAAGGGTCTTTGCTCTT |
| GPI-promoter-F: | GGATGGAGGTGAGCAATTTGG |
| GPI-promoter-R: | CACAGCTGGGAATCTTGGAC |
| PGK promoter-F: | AGACCTAACCTACCCTATCTT |
| PGK promoter-R: | AAGGGGAAAGGGCTGTTATC |
| PKM2 promoter-F: | TGCGCAACATTGTATCTGTGAATG |
| PKM2 promoter-R: | TGTGTTAGGACCGCGGAACA |
