# Supplementary material for: Phosphorylation of FOXK2 at Thr13 and Ser30 by PDK2 sustains glycolysis through a positive feedback manner in ovarian cancer
Source: Oncogene. 2024 May 11;43(26):1985–99. doi: 10.1038/s41388-024-03052-x (PMC11196215; doi:10.1038/s41388-024-03052-x)
Supplement: Supplementary file 9 — Table S4 [file 41388_2024_3052_MOESM9_ESM.docx]

**Table S4.** The databases examining FOXK2 targets-glycolytic enzymes through ChIP Seq.

| GSE84241 | | | | | |
| --- | --- | --- | --- | --- | --- |
| SYMBOL | start | end | V4 | V5 | annotation |
| PFKP | 3110374 | 3111024 | MACS_peak_988 | 31.31 | Promoter (<=1kb) |
| GAPDH | 6646593 | 6647510 | MACS_peak_1787 | 32.61 | Promoter (3-4kb) |
| PGK1 | 77359272 | 77360010 | MACS_peak_11492 | 273.26 | Promoter (<=1kb) |
| PKM | 72523192 | 72524235 | MACS_peak_3318 | 31.45 | Promoter (<=1kb) |
| LDHC | 18428712 | 18429362 | MACS_peak_1432 | 31.31 | Promoter (<=1kb) |
| PFKP | 3110374 | 3111024 | MACS_peak_988 | 33.9 | Promoter (5-6kb) |
|  |  |  |  |  |  |
| GPI | | | | | |
| CistromeDB ID | Score | Coordinate | CistromeDB ID | Score | Coordinate |
| 63241 | 0.693 | chr19:34364739-34402412 | 100620 | 0.693 | chr19:34364739-34402412 |
| 100621 | 0.694 | chr19:34364739-34402412 | 62912 | 0.039 | chr19:34353329-34400340 |
| 63743 | 0.917 | chr19:34353329-34400340 | 63744 | 0.911 | chr19:34353329-34400340 |
| 63240 | 0.826 | chr19:34353329-34400340 |  |  |  |
| HK2 | | | | | |
| CistromeDB ID | Score | Coordinate | CistromeDB ID | Score | Coordinate |
| 63240 | 0.877 | chr2:74834125-74893353 | 62913 | 0.009 | chr2:74834125-74893353 |
| 100620 | 0.986 | chr2:74835169-74893358 | 62912 | 0.159 | chr2:74834125-7489335 |
| 63744 | 0.152 | chr2:74835169-74893358 |  |  |  |
