# Supplementary material for: Phosphorylation of FOXK2 at Thr13 and Ser30 by PDK2 sustains glycolysis through a positive feedback manner in ovarian cancer
Source: Oncogene. 2024 May 11;43(26):1985–99. doi: 10.1038/s41388-024-03052-x (PMC11196215; doi:10.1038/s41388-024-03052-x)
Supplement: Supplementary file 10 — Table S5 [file 41388_2024_3052_MOESM10_ESM.docx]

**Table S5.** Phosphorylation sites of FOXK2 in three independent mass spectrometry analyses.

|  | **Protein names** | **Localization prob** | **PEP** | **Score** | **Score for localization** | **Number of Phospho (STY)** | **Amino acid** | **Intensity** | **Positions within proteins** |
| --- | --- | --- | --- | --- | --- | --- | --- | --- | --- |
| 1 | FOXK2 | 0.690825 | 1.30E-38 | 56.383 | 56.383 | 1;2 | T | 18688000 | 13 |
|  | FOXK2 | 0.666667 | 2.70E-06 | 24.919 | 18.62 | 2 | S | 4462400 | 9 |
|  | FOXK2 | 0.666667 | 0.00011753 | 19.622 | 18.62 | 2 | S | 4462400 | 30 |
|  | FOXK2 | 0.24191 | 0.0007788 | 24.459 | 17.076 |  | S | 0 | 384 |
|  | FOXK2 | 0.226857 | 0.0007788 | 24.459 | 24.459 |  | S | 0 | 373 |
|  | FOXK2 | 0.226857 | 0.0007788 | 24.459 | 24.459 |  | S | 0 | 381 |
|  | FOXK2 | 0.226857 | 0.0007788 | 24.459 | 24.459 |  | S | 0 | 385 |
| 2 | FOXK2 | 0.999208 | 4.63E-22 | 180.23 | 82.482 | 1;2 | T | 2.514E+10 | 13 |
|  | FOXK2 | 1 | 2.90E-15 | 160.5 | 160.5 | 1;2 | S | 1.6818E+10 | 30 |
|  | FOXK2 | 0.999678 | 9.96E-15 | 194.19 | 194.19 | 1 | S | 1756600000 | 61 |
|  | FOXK2 | 1 | 1.53E-05 | 99.846 | 47.281 | 1 | T | 411770000 | 84 |
|  | FOXK2 | 1 | 0.00069333 | 93.649 | 93.649 | 2 | S | 135050000 | 168 |
|  | FOXK2 | 1 | 8.81E-21 | 270.79 | 93.649 | 1;2 | S | 2059500000 | 170 |
|  | FOXK2 | 0.67394 | 5.55E-06 | 96.098 | 96.098 | 1 | S | 260440000 | 195 |
|  | FOXK2 | 0.466471 | 3.16E-15 | 156.73 | 156.73 |  | T | 0 | 201 |
|  | FOXK2 | 0.999942 | 7.97E-09 | 131.79 | 131.79 | 1 | S | 0 | 230 |
|  | FOXK2 | 1 | 9.24E-15 | 156.9 | 145.17 | 1 | S | 1438900000 | 239 |
|  | FOXK2 | 0.999716 | 5.35E-15 | 165.96 | 143.36 | 1;2 | S | 7982900000 | 248 |
|  | FOXK2 | 0.999281 | 1.32E-10 | 143.36 | 143.36 | 2 | S | 554510000 | 252 |
|  | FOXK2 | 0.555184 | 1.13E-09 | 131.97 | 131.97 | 1 | S | 6592600000 | 257 |
|  | FOXK2 | 0.182809 | 0.0255848 | 45.527 | 45.527 |  | S | 0 | 262 |
|  | FOXK2 | 0.999995 | 6.66E-20 | 181.37 | 161.55 | 1;2;3 | S | 1.0885E+10 | 373 |
|  | FOXK2 | 0.860875 | 0.00101789 | 71.297 | 53.728 | 3 | S | 307070000 | 381 |
|  | FOXK2 | 0.886222 | 3.16E-26 | 204.47 | 204.47 | 1;2;3 | S | 1399000000 | 384 |
|  | FOXK2 | 0.604425 | 0.00101789 | 71.297 | 46.926 | 3 | S | 307070000 | 385 |
|  | FOXK2 | 0.998253 | 6.37E-15 | 157.59 | 128.47 | 1;2;3 | T | 9535200000 | 389 |
|  | FOXK2 | 0.98752 | 8.31E-27 | 219.52 | 63.913 | 1;2;3 | S | 1.3867E+10 | 392 |
|  | FOXK2 | 0.997662 | 3.94E-23 | 176.31 | 63.913 | 2;3 | S | 7710700000 | 394 |
|  | FOXK2 | 1 | 3.08E-10 | 159.02 | 159.02 | 1;2;3 | S | 5819700000 | 398 |
|  | FOXK2 | 0.999985 | 1.82E-10 | 159.67 | 159.67 | 1 | S | 267120000 | 424 |
|  | FOXK2 | 0.999976 | 6.16E-11 | 167.63 | 167.63 | 1 | S | 1081700000 | 428 |
|  | FOXK2 | 0.537113 | 0.00056462 | 82.342 | 82.342 | 1 | T | 144050000 | 551 |
|  | FOXK2 | 0.999999 | 1.08E-32 | 217.55 | 217.55 | 1 | S | 9934900000 | 599 |
|  | FOXK2 | 1 | 6.39E-23 | 217.09 | 217.09 | 1 | T | 5023400000 | 634 |
| 3 | FOXK2 | 0.999999 | 3.74E-05 | 86.577 | 86.577 | 1 | T | 543290000 | 634 |
|  | FOXK2 | 0.99714 | 6.14E-05 | 56.111 | 56.111 | 2 | S | 127320000 | 30 |
|  | FOXK2 | 0.961759 | 6.87E-13 | 91.398 | 91.398 | 1;2 | T | 318730000 | 13 |
